# Characterization and phylogenetic implications of newly sequenced mitogenomes of Five *Mileewa* and *Processina* species from China (Hemiptera: Cicadellidae: Mileewinae)

**DOI:** 10.1038/s41598-022-25376-y

**Published:** 2022-12-02

**Authors:** Hongli He, Bin Yan, Xiaofei Yu, Maofa Yang

**Affiliations:** 1 Institute of Entomology of Guizhou University, Guizhou Provincial Key Laboratory for Agricultural Pest Management of the Mountainous Region, Guiyang, 550025 Guizhou China; 2 College of Tobacco Sciences of Guizhou University, Guiyang, 550025 Guizhou China

**Keywords:** Entomology, Taxonomy, Comparative genomics

## Abstract

To accumulate evidence of the phylogeny of Mileewinae and the relationships among *Mileewa*, *Processina,* and *Ujna* genera, we sequenced the complete mitochondrial genomes of four *Mileewa* spp., namely, *Mileewa mira*, *Mileewa lamellata*, *Mileewa sharpa,* and *Mileewa amplimacula*. The first complete mitogenome of the genus *Processina* (*P. sexmaculata*), established by Yang, Deitz & Li from China and comprising five species, was also sequenced in this study. Annotation showed that the five mitogenomes were 14787 –15436 bp in length, and all harbored 37 typical genes. The AT content of the five mitogenomes ranged from 78.3% to 80.2%, which was similar to that of other sequenced Mileewinae species. For protein-coding genes (PCGs), ATN was the start codon, while atp8 and nad5 genes were initiated with TTG, and a great majority of them used TAA or TAG as stop codons, whereas cox2 and nad1 ended with an incomplete codon T–. All tRNAs had a typical cloverleaf secondary structure, except for trnS1, which had a reduced dihydrouridine arm. We further used 59 Membracoidea species and two outgroups to reconstruct phylogenetic trees based on 13 PCGs under an independent partition model with Bayesian inference and Maximum-likelihood methods. Among these two trees, each of the subfamilies Cicadellinae, Typhlocybinae, and Mileewinae were recovered as a monophyletic group with high support values, suggesting that Typhlocybinae was more ancient than Mileewinae and Cicadellinae. Within the Mileewinae subfamily, all species maintained the same relationships and topologies according to both the BI and ML analyses (PP > 0.8, BS > 83) as follows: (*M. sharpa* + (*U. puerana* + ((*M. ponta* + (*M. mira* + *M. lamellata*)) + ((*M. albovittata* + (*M. margheritae* + *M. amplimacula*)) + (*M. rufivena* + (*P. sexmaculata* + *M. alara*)))))), and the monophyly of the genera *Processina, Mileewa* and *Ujna* were not supported. This study further enriches the Mileewinae mitogenome database and will contribute to future research on the systematics, evolution, and classification of this group.

## Introduction

The small leafhopper subfamily Mileewinae has approximately 160 described species worldwide. The subfamily contains small and medium-sized, slender, usually darkly pigmented species that inhabit wet tropical forests worldwide, where they usually occur on herbaceous vegetation in the understory. With the changing taxonomic status of the subfamily Mileewinae, there is some controversy regarding the relationship between this subfamily and both Cicadellinae and Typhlocybinae^1–5^. The subfamily Mileewinae includes four tribes: Makilingiini (Philippines and Thailand), Mileewini (Old and New World), Tinteromini (New World), and Tungurahualini (New World)^6,7^. All species of this subfamily in China belong to Mileewini and now comprise four genera: *Mileewa* Distant, *Ujna* Distant^8^, *Processina* Yang, Deitz & Li^9^, and *Anzihelus* Yan & Yang—a new genus established in 2021^10^ which is can be distinguished by its elevated head above the pronotum and the male Xth segment (basal anal tube segment) with single caudal process. A great majority of Chinese Mileewini species belong to the genera *Mileewa,* which has 56 species, *Ujna,* which has eight species, and *Processina*, which has five species that are only recorded in China^11^.

*Mileewa* is a widespread and large genus of the tribe Mileewini established with *Mileewa margheritae* by Distant (1908). The genus has more than 80 valid species globally that have a dark appearance dorsally and forewing that is usually truncated or concave apically and sometimes distinctly expanded from base to apex. Moreover, in our recent study, seven species were newly recorded in this genus from China^11–13^. The genus *Processina* was completely established in China by Yang, Deitz & Li in 2005 and contains five species. *Processina dashahensis* (Yang et Li, 2005) is one of these species that was recorded in the Dashahe Natural Reserve of Guizhou Province. The other species originated from several places, such as Taiwan, Yunnan, Guizhou, and Sichuan. The traditional classification difference between the genera *Mileewa* and *Ujna* is that the head width is narrower than the pronotum or subequal to the pronotum width; the forewing apex is truncated or emarginated in *Mileewa* yet rounded in *Ujna*; and male style have long setae near their middle section in *Mileewa* versus an absent long setae in *Ujna*. In contrast, the genus *Processina* is distinguished from the other two genera mainly by its male style with a straight apical portion, rounded apex, and dense setae at the apex^14^. However, some ambiguous features make it difficult to distinguish between species in traditional classification studies. Therefore, there is an urgent need to increase our knowledge on the mitochondrial genomes of Mileewinae species and to use these molecular data to determine the phylogenetic relationships among other subfamilies and within this small group.

The insect mitochondrial genomes are mostly 14.5–17 kb double-stranded circular molecules comprising 37 typical genes: 13 protein-coding genes (PCGs), two ribosomal RNA genes (rRNAs: s-rRNA; l-rRNA), 22 transfer RNA genes (tRNAs), and a control region (CR)^15–17^. To date, six Mileewinae species from China have been sequenced, and these sequences have been deposited in the NCBI database: *Mileewa rufivena* (MZ326689)^18^, *Mileewa ponta* (MT497465)^19^, *Mileewa margheritae* (MT483998)^20^, *Mileewa albovittata* (MK138358)^21^, *Mileewa alara* (MW533151)^22^ and *Ujna puerana* (MZ326688)^18^. They are all from the genera *Ujna* and *Mileewa*, while sequences of *Processina* species have not been deposited in NCBI. In this study, we sequenced and annotated the complete mitogenomes of five Mileewinae species: one from the genus *Processina*, and others from *Mileewa*. Furthermore, we analyzed the characteristics of these mitogenomes, including nucleotide composition, tRNA secondary structure, codon usage, gene overlaps, intergenic spacer, and CRs. The mitochondrial genome data in this study can help us understand the phylogeny and evolution of Mileewinae. The growing number of studies on the complete mitogenomes of Mileewinae will yield more evidence, which can render a better understanding of the taxonomic status of the subfamily Mileewinae among other Cicadellidae subfamilies.

## Materials and methods

### Sample collection and taxonomic identification

Specimens of adult male *M. mira*, *M. lamellata*, *M. sharpa*, *M. amplimacula,* and *P. sexmaculata* were collected from China (Table S1). All specimens were collected, immediately preserved in 100% ethanol, and stored at −20 °C in a laboratory freezer prepared for DNA extraction. The species used in this study (males) were identified by Maofa-Yang based on morphological characteristics, especially male genitalia, according to the taxonomy system described by Dietrich (2011)^6^.

### DNA extraction and sequencing

After identifying the species, total genomic DNA was extracted from the head and thorax tissues of five single adult species, using the Tissue and Blood Genome DNA Extraction Kit (Qiagen, Hilden, Germany), according to the manufacturer’s protocols. Voucher DNA was stored at −20 °C, while the external genitalia were kept in glycerol. Both were deposited at the Institute of Entomology, Guizhou University, Guiyang, China (GUGC). The five new mitogenomes were subjected to next-generation sequencing on the Illumina NovaSeq6000 platform (Berry Genomics, Beijing, China) with a paired-end 150 sequencing strategy.

### Mitogenomes assembly, annotation, and analysis

The clean sequence reads were assembled using Getorganelle 1.7.5^23^ based on the *Mileewa rufivena* mitochondrial genome sequence from GenBank (Accession number: MZ326689.1)^18^. The five mitogenomes were initially annotated using MitoZ 2.4-alpha with invertebrate mitochondrial genetic codes^24^. The annotated results from MitoZ were then imported into Geneious Prime 2021.1 software for further editing. We used the MITOS (http://mitos.bioinf.uni-leipzig.de/index.py) web server^25^ with invertebrate genetic codes to further revise the tRNA and PCG locations and generate the secondary structures of tRNAs and the ARWEN program^26^ to check the MITOS results for tRNA secondary structures and locations. ORF finders in Geneious Prime were also used to annotate PCGs using the invertebrate genetic code. l-rRNA genes were defined according to adjacent tRNA genes (trnL1 and trnV), whereas s-rRNA genes were located by comparison with other homologous Cicadellidae species s-rRNA genes. Mitogenomic circular maps were generated using OGDRAW website (https://chlorobox.mpimp-golm.mpg.de/OGDraw.html)^27^. The nucleotide composition and relative synonymous codon usage (RSCU) were calculated using Phylosuite v1.2.2 software^28^. Strand asymmetry was calculated according to the formula AT skew = [A − T]/[A + T] and GC skew = [G − C]/[G + C]^29^. Tandem repeats in the CR were recognized using the Tandem Repeats Finder program (https://tandem.bu.edu/trf/trf.basic.submit.html)^30^. Finally, the five newly sequenced mitogenomes of Mileewinae were submitted to GenBank with a tbl file using GB2sequin (https://chlorobox.mpimp-golm.mpg.de/GenBank2Sequin.html)^31^ under the accession numbers ON464171–ON464175.

### Phylogenetic analyses

For phylogenetic analyses, mitochondrial genomes of 61 species belonging to 17 subfamilies were selected. Of them, mitogenomes of 59 Membracoidea species (50 leafhopper, 4 treehopper, and 5 newly sequenced species) comprised the ingroup, and those of two species—*Callitettix braconoides* (NC_025497)^32^ and *Tettigades auropilosa* (KM000129)—were used as outgroups. The detailed information and accession numbers for these mitogenomes are listed in Table S2.

For the five newly sequenced species, each PCG in the mitogenome was manually extracted using the Geneious Prime 2021.1 program. Thirteen PCG sequences were aligned in batches with MAFFT^33^ using the’–auto’ strategy and codon alignment mode. The alignments were refined using the codon-aware program MACSE v. 2.03^34^, which preserves the reading frame and allows the incorporation of sequencing errors or sequences with frameshifts. Ambiguously aligned fragments of 13 alignments were removed in batches using Gblocks^35^. The alignments of each individual gene were concatenated using the Phylosuite^28^ program. The best partitioning scheme and evolutionary models for 39 predefined partitions were selected using PartitionFinder2^36^ with a greedy algorithm^37^ and Akaike Information Criterion (AIC) in Bayesian inference (BI) analyses and auto-generated in IQ-TREE^38^ with Bayesian Information Criterion (BIC) in maximum-likelihood (ML) analyses. Detailed model information is shown in Table S3. BI analysis was inferred using MrBayes 3.2.6^39^ with four chains under an independent partition model (two parallel runs, 10,466,900 generations) with sampling every 100 generations, in which the initial 25% of sampled data were discarded as burn-in, and the remaining trees were used to generate a consensus tree and calculate Bayesian posterior probability (PP) values after the average standard deviation of split frequencies was < 0.01. ML analysis was performed using the IQ-TREE program^38^ under an edge-linked partition model for 1000 replicates of ultrafast^40^ bootstraps and generated bootstrap support (BS). BI and ML trees were viewed and edited using the iTOL online tool (https://itol.embl.de/)^41^.

## Results

### Genome organization and composition

**Figure 1:**
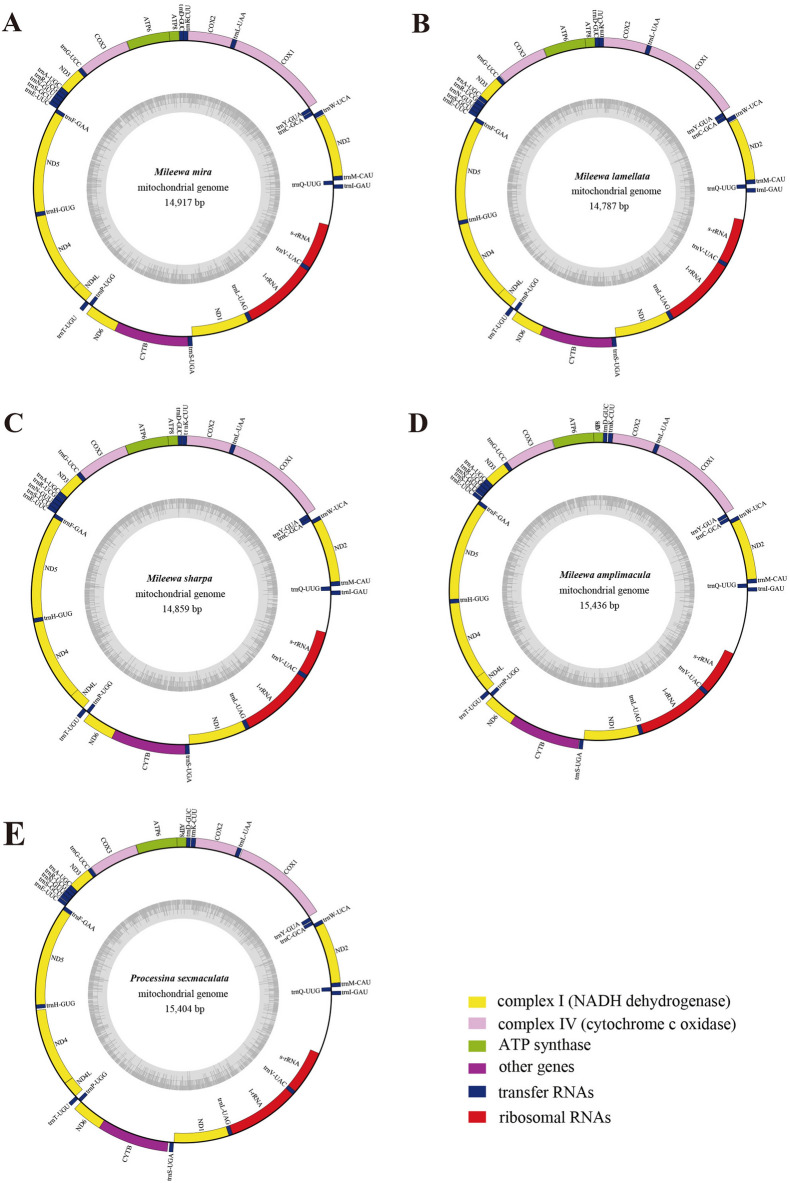
Circular maps of the mitogenomes of *Mileewa mira* (**A**), *Mileewa lamellata* (**B**), *Mileewa sharpa* (**C**), *Mileewa amplimacula* (**D**), and *Processina sexmaculata* (**E**). Genes are shown in different color blocks. Color blocks outside the circle indicates that the genes are located on the heavy strand (H-strand); color blocks within the circle indicates that the genes are located on the light strand (L-strand).

The five new complete mitogenomes were identified as circular double-stranded molecules with the length of 14787–15436 bp for *Mileewa mira*, *M. lamellata*, *M. sharpa*, *M. amplimacula, P. sexmaculata* (ON464171–ON464175), respectively. Detailed annotations and circular maps are presented in Fig. 1 and Table 1. The species were medium length compared to other published Mileewinae species^18–22^. Each of the five newly sequenced mitogenomes contained 37 typical mitochondrial genes: 13 PCGs, two rRNA genes, 22 tRNA genes, and a CR (Fig. 1, Table 1). Regarding gene arrangement, all five mitogenome sequences displayed identical gene orders, consistent with previously published mitogenomes of Cicadellidae^18,42,43^. Of these 37 genes, 23 (9 PCGs and 14 tRNAs) were located on the heavy (H) strand, whereas 14 (4 PCGs, 8 tRNAs, and 2 rRNAs) were located on the light (L) strand.

**Table 1:** Organization of the four *Mileewa* and one *Processina* species mitochondrial genomes.

Gene	Position		Size(bp)	Intergenic nucleotides	Codon		Strand
	From	To			Start	Stop	
***Mileewa mira*/*Mileewa lamellata*/*Mileewa sharpa*/*Mileewa amplimacula*/*Processina sexmaculata***							
trnI	1/1/1/1/1	64/67/66/63/63	64/67/66/63/63				H/H/H/H/H
trnQ	62/65/69/61/61	128/131/136/129/128	67/67/68/69/68	−3/−3/2/−3/−3			L/L/L/L/L
trnM	129/132/137/130/129	193/197/203/195/193	65/66/67/66/65				H/H/H/H/H
nad2	194/198/204/196/194	1162/1166/1172/1161/1162	969/969/969/966/969		ATA/ATA/ATA/ATA/ATA	TAA/TAA/TAA/TAA/TAA	H/H/H/H/H
trnW	1174/1178/1171/1160/1162	1245/1246/1231/1222/1224	72/69/61/63/63	11/11/−2/−2/−1			H/H/H/H/H
trnC	1238/1239/1224/1215/1217	1300/1300/1285/1276/1279	63/62/62/62/63	−8/−8/−8/−8/−8			L/L/L/L/L
trnY	1307/1304/1285/1286/1289	1370/1366/1348/1348/1354	64/63/64/63/66	6/3/−1/9/9			L/L/L/L/L
cox1	1379/1374/1347/1350/1369	2914/2909/2888/2885/2904	1536/1536/1542/1536/1536	8/7/−2/1/14	ATG/ATG/ATG/ATG/ATG	TAA/TAA/TAA/TAG/TAA	H/H/H/H/H
trnL2	2916/2911/2884/2886/2918	2982/2978/2948/2951/2984	67/68/65/66/67	1/1/−5/–/13			H/H/H/H/H
cox2	2983/2979/2949/2952/2985	3659/3655/3629/3631/3658	677/677/681/680/674		ATG/ATA/ATA/ATG/ATA	TA/TA/TAA/TA/TA	H/H/H/H/H
trnK	3660/3656/3630/3632/3659	3731/3727/3699/3703/3729	72/72/70/72/71				H/H/H/H/H
trnD	3730/3726/3700/3718/3738	3792/3789/3761/3780/3803	63/64/62/63/66	−2/−2/–/14/8			H/H/H/H/H
atp8	3793/3790/3762/3781/3804	3945/3942/3914/3933/3956	153/153/153/153/153		TTG/TTG/TTG/TTG/TTG	TAA/TAA/TAA/TAA/TAA	H/H/H/H/H
atp6	3939/3936/3908/3927/3950	4592/4589/4561/4580/4603	654/654/654/654/654	−7/−7/−7/−7/−7	ATG/ATG/ATG/ATG/ATG	TAA/TAA/TAA/TAA/TAA	H/H/H/H/H
cox3	4596/4593/4562/4588/4606	5375/5372/5341/5367/5385	780/780/780/780/780	3/3/–/7/2	ATG/ATG/ATG/ATG/ATG	TAA/TAA/TAA/TAA/TAA	H/H/H/H/H
trnG	5378/5382/5341/5367/5390	5440/5447/5403/5429/5451	63/66/63/63/62	2/9/−1/−1/4			H/H/H/H/H
nad3	5441/5448/5404/5430/5452	5794/5801/5754/5783/5805	354/354/351/354/354		ATT/ATA/ATC/ATT/ATT	TAG/TAA/TAG/TAA/TAG	H/H/H/H/H
trnA	5793/5801/5753/5783/5804	5854/5862/5814/5845/5864	62/62/62/63/61	−2/−1/−2/−1/−2			H/H/H/H/H
trnR	5855/5868/5817/5845/5864	5919/5929/5879/5911/5927	65/62/63/67/64	–/5/2/−1/−1			H/H/H/H/H
trnN	5917/5927/5878/5909/5925	5980/5990/5944/5973/5990	64/64/67/65/66	−3/−3/−2/−3/−3			H/H/H/H/H
trnS1	5980/5990/5944/5973/5990	6046/6056/6010/6038/6055	67/67/67/66/66	−1/−1/−1/−1/−1			H/H/H/H/H
trnE	6046/6056/6010/6048/6060	6108/6119/6071/6112/6123	63/64/62/65/64	−1/−1/−1/9/4			H/H/H/H/H
trnF	6109/6120/6071/6113/6125	6171/6184/6132/6176/6188	63/65/62/64/64	–/–/−1/–/1			L/L/L/L/L
nad5	6171/6184/6132/6176/6192	7844/7857/7804/7849/7865	1674/1674/1673/1674/1674	−1/−1/−1/−1/3	TTG/TTG/TTG/TTG/TTG	TAA/TAA/TA/TAA/TAA	L/L/L/L/L
trnH	7845/7858/7805/7850/7866	7910/7920/7868/7915/7930	66/63/64/66/65				L/L/L/L/L
nad4	7911/7921/7868/7919/7954	9233/9241/9190/9235/9273	1323/1321/1323/1317/1320	–/–/−1/3/23	ATG/ATG/ATG/ATG/ATA	TAA/T/TAA/TAA/TAA	L/L/L/L/L
nad4L	9227/9235/9184/9229/9273	9502/9510/9456/9504/9548	276/276/273/276/276	−7/−7/−7/−7/−1	ATG/ATG/ATG/ATT/ATG	TAA/TAA/TAG/TAA/TAA	L/L/L/L/L
trnT	9505/9513/9459/9507/9551	9571/9579/9525/9573/9614	67/67/67/67/64	2/2/2/2/2			H/H/H/H/H
trnP	9572/9580/9526/9574/9615	9635/9643/9586/9639/9678	64/64/61/66/64				L/L/L/L/L
nad6	9638/9646/9589/9642/9681	10133/10137/10080/10136/10172	496/492/492/495/492	2/2/2/2/2	ATT/ATT/ATC/ATC/ATA	T/TAA/TAA/TAA/TAA	H/H/H/H/H
cytb	10122/10130/10073/10129/10165	11258/11266/11209/11265/11301	1137/1137/1137/1137/1137	−12/−8/−8/−8/−8	ATG/ATG/ATG/ATG/ATG	TAA/TAA/TAG/TAA/TAA	H/H/H/H/H
trnS2	11257/11265/11208/11270/11326	11324/11332/11272/11334/11392	68/68/65/65/67	−2/−2/−2/4/24			H/H/H/H/H
nad1	11326/11334/11274/11337/11394	12253/12261/12204/12264/12321	928/928/931/928/928	1/1/1/2/1	ATT/ATT/ATA/ATT/ATA	T/T/T/T/T	L/L/L/L/L
trnL1	12254/12262/12205/12265/12322	12319/12327/12272/12331/12386	66/66/68/67/65				L/L/L/L/L
rrnL	12320/12328/12273/12332/12387	13515/13523/13452/13548/13593	1196/1196/1180/1217/1207				L/L/L/L/L
trnV	13516/13524/13453/13549/13594	13578/13586/13518/13617/13658	63/63/66/69/65				L/L/L/L/L
rrnS	13579/13587/13519/13618/13659	14326/14341/14258/14374/14408	748/755/740/757/750				L/L/L/L/L
control region	14327/14342/14259/14375/14409	14917/14787/14859/15436/15404	591/446/601/1062/996				H/H/H/H/H
–: none							

The nucleotide composition of these five species was listed in Table 2. The AT nucleotide content of the five mitogenomes ranged from 78.3% (*P. sexmaculata*) to 80.2% (*M. lamellata*), whereas the GC nucleotide content ranged from 19.8% (*M. lamellata*) to 21.6% (*M. sharpa* and *P. sexmaculata*), revealing a significant AT bias. The highest and lowest AT contents were found in the CR (83.3%–86.6%) and PCGs (77.1%–79.6%), and the order was CR > rRNAs > tRNAs > PCGs. All five whole mitogenomes exhibited a positive AT skew (0.057–0.098), indicating that the A nucleotide was more prevalent than the T nucleotide, and a negative GC skew (−0.170 to −0.101), indicating that the nucleotide percentage of C was larger than that of G.

**Table 2:** Nucleotide composition and skewness of the four *Mileewa* and one *Processina* species mitochondrial genomes.

Regions	Size (bp)	T(U)	C	A	G	AT(%)	GC(%)	GT(%)	AT skew	GC skew
***Mileewa mira*/*Mileewa lamellata*/*Mileewa sharpa*/*Mileewa amplimacula*/*Processina sexmaculata***										
PCGs	10953/10947/10956/10947/10944	45.8/45.8/45/44.4/44.2	10.4/9.9/10.8/11/11.3	32.4/33.8/32.4/32.9/32.9	11.4/10.5/11.8/11.7/11.6	78.2/79.6/77.4/77.3/77.1	21.8/20.4/22.6/22.7/22.9	57.2/56.3/56.8/56.1/55.8	−0.172/−0.15/−0.164/−0.147/−0.147	0.046/0.029/0.047/0.034/0.014
1st codon position	3651/3649/3652/3649/3648	39.1/38.5/38.1/37.1/37.4	9.7/9.6/10/10.2/10.7	36.2/37.3/35.4/37.4/36.2	15/14.6/16.5/15.3/15.7	75.3/75.8/73.5/74.5/73.6	24.7/24.2/26.5/25.5/26.4	54.1/53.1/54.6/52.4/53.1	−0.039/−0.016/−0.037/0.004/−0.016	0.214/0.206/0.242/0.2/0.189
2nd codon position	3651/3649/3652/3649/3648	49.3/49.2/48.7/48.8/48.6	16.1/16.1/16.8/16/16.8	20.8/21/21.1/21.4/20.6	13.9/13.6/13.4/13.8/14	70.1/70.2/69.8/70.2/69.2	30/29.7/30.2/29.8/30.8	63.2/62.8/62.1/62.6/62.6	−0.407/−0.401/−0.396/−0.391/−0.405	−0.073/−0.085/−0.113/−0.074/−0.09
3rd codon position	3651/3649/3652/3649/3648	48.9/49.7/48.2/47.2/46.7	5.5/3.9/5.5/6.7/6.3	40.2/43.1/40.6/40.1/41.9	5.4/3.3/5.7/6.1/5.1	89.1/92.8/88.8/87.3/88.6	10.9/7.2/11.2/12.8/11.4	54.3/53/53.9/53.3/51.8	−0.098/−0.07/−0.086/−0.081/−0.055	−0.008/−0.095/0.017/−0.043/−0.108
atp6	654/654/654/654/654	44.8/42.8/43.9/42/40.1	11.3/11.3/12.4/12.8/13.9	34.7/36.5/34.7/35.6/36.4	9.2/9.3/9/9.5/9.6	79.5/79.3/78.6/77.6/76.5	20.5/20.6/21.4/22.3/23.5	54/52.1/52.9/51.5/49.7	−0.127/−0.079/−0.117/−0.083/−0.048	−0.104/−0.096/−0.157/−0.151/−0.182
atp8	153/153/153/153/153	42.5/37.3/45.1/39.2/35.3	9.8/9.8/9.2/9.2/9.8	41.8/49.7/37.9/46.4/49.7	5.9/3.3/7.8/5.2/5.2	84.3/87/83/85.6/85	15.7/13.1/17/14.4/15	48.4/40.6/52.9/44.4/40.5	−0.008/0.143/−0.087/0.084/0.169	−0.25/−0.5/−0.077/−0.273/−0.304
cox1	1536/1536/1542/1536/1536	40.7/39.5/41.5/38.9/38.5	13.3/13.2/12.6/15.2/14.2	31.1/33.6/31.4/30.2/32.8	15/13.8/14.5/15.7/14.5	71.8/73.1/72.9/69.1/71.3	28.3/27/27.1/30.9/28.7	55.7/53.3/56/54.6/53	−0.134/−0.08/−0.139/−0.125/−0.079	0.06/0.024/0.067/0.015/0.011
cox2	677/677/681/680/674	40.9/39.6/40.4/35.9/36.5	11.8/12.9/11.9/15.3/15.6	36.3/38.7/37.2/38.7/38.6	10.9/8.9/10.6/10.1/9.3	77.2/78.3/77.6/74.6/75.1	22.7/21.8/22.5/25.4/24.9	51.8/48.5/51/46/45.8	−0.059/−0.011/−0.042/0.037/0.028	−0.039/−0.184/−0.059/−0.202/−0.25
cox3	780/780/780/780/780	43.3/39.4/40.8/38.8/39.5	12.4/13.6/12.6/13.6/13.8	31.4/35.4/34.1/35.1/34.7	12.8/11.7/12.6/12.4/11.9	74.7/74.8/74.9/73.9/74.2	25.2/25.3/25.2/26/25.7	56.1/51.1/53.4/51.2/51.4	−0.16/−0.053/−0.089/−0.05/−0.064	0.015/−0.076/0/−0.044/−0.075
cytb	1137/1137/1137/1137/1137	39.1/40.5/38.9/39/36.9	15.1/13/15.6/14.7/16.3	34.3/36/33.7/34.9/35.6	11.5/10.6/11.9/11.4/11.2	73.4/76.5/72.6/73.9/72.5	26.6/23.6/27.5/26.1/27.5	50.6/51.1/50.8/50.4/48.1	−0.065/−0.059/−0.072/−0.055/−0.018	−0.135/−0.104/−0.135/−0.125/−0.186
nad1	928/928/931/928/928	51.4/52.7/49.8/52.4/51.1	8.8/7.8/8.6/7.7/8.1	24.8/26.8/26.2/25.2/24.9	15/12.7/15.4/14.8/15.9	76.2/79.5/76/77.6/76	23.8/20.5/24/22.5/24	66.4/65.4/65.2/67.2/67	−0.349/−0.325/−0.311/−0.35/−0.345	0.258/0.242/0.283/0.317/0.327
nad2	969/969/969/966/969	46.7/45.8/47.5/44.5/43.9	7.5/8.4/8.2/8.8/9.6	37.2/38.6/34.1/38.4/38.4	8.6/7.2/10.3/8.3/8.2	83.9/84.4/81.6/82.9/82.3	16.1/15.6/18.5/17.1/17.8	55.3/53/57.8/52.8/52.1	−0.114/−0.086/−0.165/−0.074/−0.066	0.064/−0.073/0.117/−0.03/−0.081
nad3	354/354/351/354/354	44.9/45.5/44.7/41/42.4	9.9/9.9/10/12.1/12.1	37.6/37.3/36.5/39.3/38.7	7.6/7.3/8.8/7.6/6.8	82.5/82.8/81.2/80.3/81.1	17.5/17.2/18.8/19.7/18.9	52.5/52.8/53.5/48.6/49.2	−0.089/−0.099/−0.102/−0.021/−0.045	−0.129/−0.148/−0.061/−0.229/−0.284
nad4	1323/1321/1323/1317/1320	52.9/54.5/50.9/52.5/52.6	8.1/6.7/8.9/7.9/8	27.6/27.3/27.6/26.7/26.1	11.4/11.5/12.5/12.9/13.4	80.5/81.8/78.5/79.2/78.7	19.5/18.2/21.4/20.8/21.4	64.3/66/63.4/65.4/66	−0.315/−0.333/−0.297/−0.325/−0.337	0.171/0.261/0.169/0.241/0.255
nad4L	276/276/273/276/276	51.8/53.6/55.7/55.1/55.4	5.8/4.3/7/3.3/4.7	29.7/31.2/24.5/29.7/26.8	12.7/10.9/12.8/12/13	81.5/84.8/80.2/84.8/82.2	18.5/15.2/19.8/15.3/17.7	64.5/64.5/68.5/67.1/68.4	−0.271/−0.265/−0.388/−0.299/−0.348	0.373/0.429/0.296/0.571/0.469
nad5	1674/1674/1673/1674/1674	50.1/52.3/48.8/50.3/52.3	8.5/7/8.8/7.8/7.7	30.5/30.3/30.7/30.1/27.8	10.9/10.5/11.7/11.8/12.1	80.6/82.6/79.5/80.4/80.1	19.4/17.5/20.5/19.6/19.8	61/62.8/60.5/62.1/64.4	−0.243/−0.266/−0.227/−0.251/−0.306	0.123/0.199/0.137/0.207/0.223
nad6	496/492/492/495/492	41.3/40.9/36.6/38.2/38.8	8.9/9.1/11.4/9.9/10.8	43.8/44.3/46.1/45.3/45.3	6/5.7/5.9/6.7/5.1	85.1/85.2/82.7/83.5/84.1	14.9/14.8/17.3/16.6/15.9	47.3/46.6/42.5/44.9/43.9	0.028/0.041/0.115/0.085/0.077	−0.189/−0.233/−0.318/−0.195/−0.359
l-rRNA	1196/1196/1180/1217/1207	46.2/46.1/48.2/44.9/47.7	6.6/6.9/6.4/7.3/7.1	36/35.8/33.3/36.7/33.6	11.3/11.2/12/11.1/11.5	82.2/81.9/81.5/81.6/81.3	17.9/18.1/18.4/18.4/18.6	57.5/57.3/60.2/56/59.2	−0.124/−0.126/−0.183/−0.1/−0.173	0.262/0.235/0.303/0.205/0.236
s-rRNA	748/755/740/757/750	44.3/45.8/47/43.2/45.3	8.2/7/7.3/8.9/7.5	35/34.4/33.2/35.1/34.3	12.6/12.7/12.4/12.8/12.9	79.3/80.2/80.2/78.3/79.6	20.8/19.7/19.7/21.7/20.4	56.9/58.5/59.4/56/58.2	−0.116/−0.142/−0.172/−0.103/−0.139	0.213/0.289/0.26/0.183/0.268
rRNAs	1944/1951/1920/1974/1957	45.4/46/47.8/44.2/46.8	7.2/7/6.8/7.9/7.3	35.6/35.3/33.3/36.1/33.9	11.8/11.8/12.2/11.8/12.1	81/81.3/81.1/80.3/80.7	19/18.8/19/19.7/19.4	57.2/57.8/60/56/58.9	−0.121/−0.132/−0.179/−0.101/−0.16	0.241/0.257/0.286/0.196/0.249
tRNAs	1438/1439/1422/1440/1429	39.9/39.9/39.3/40.3/38.9	7.6/7.7/8.3/8.2/8.1	41/41.5/40.6/40.1/41.5	11.4/10.9/11.7/11.3/11.5	80.9/81.4/79.9/80.4/80.4	19/18.6/20/19.5/19.6	51.3/50.8/51/51.6/50.4	0.014/0.02/0.017/−0.003/0.032	0.197/0.172/0.172/0.16/0.171
control region	591/446/601/1062/996	42.3/40.8/42.1/42.2/44.1	8/6.1/7.3/6.9/8	42.6/45.7/44.1/44.4/39.2	7.1/7.4/6.5/6.5/8.7	84.9/86.5/86.2/86.6/83.3	15.1/13.5/13.8/13.4/16.8	49.4/48.2/48.6/48.7/52.8	0.004/0.057/0.023/0.025/−0.059	−0.06/0.096/−0.058/−0.03/0.042
Full genome	14917/14787/14859/15436/15404	37.3/36.7/36.7/36.2/35.3	11.5/11.3/12/12.2/12.6	41.8/43.5/41.7/42.4/43	9.4/8.5/9.6/9.2/9	79.1/80.2/78.4/78.6/78.3	20.9/19.8/21.6/21.4/21.6	46.7/45.2/46.3/45.4/44.3	0.057/0.085/0.064/0.079/0.098	−0.101/−0.142/−0.111/−0.139/−0.17

PCGs: Protein-coding genes, rRNAs: ribosomal RNA genes, tRNAs: transfer RNA genes.

### PCGs and codon usage

Among the five new mitogenomes, the total lengths of the 13 PCGs were 10953 bp in *M. mira*, 10947 bp in *M. lamellata*, 10956 bp in *M. sharpa*, 10947 bp in *M. amplimacula*, and 10944 bp in *P. sexmaculata* (Table 2). Each of the PCG sequence exhibited a comparable size, with the nad5 gene being the longest (1674 bp, except 1673 bp for *M. sharpa*) and atp8 being the shortest (153 bp). Of the 13 PCGs, 9 PCGs (cox1, cox2, cox3, atp6, atp8, nad2, nad3, nad6, and cytb) were located on the H-strand, and the remaining four (nad1, nad4, nad4L, and nad5) on the L-strand (Fig. 1, Table 1). In five newly annotated mitogenomes, most PCGs were initiated with the typical codon ATN (ATA, ATG, ATC, and ATT), while atp8 and nad5 were initiated with the codon TTG, similar to a previous report^18–22^. The majority of PCGs terminated with the complete stop codons TAA and TAG; however, some PCGs used an incomplete stop codon T– (TA or T, as shown in Table 1), such as nad1 and cox2. This phenomenon is common in invertebrate mitogenomes and can be completed by post-transcriptional polyadenylation^17,44^. Furthermore, nad2, atp8, atp6, and cox3 genes of all the five species had the same start and stop codons. The AT content of *M. mira, M. lamellata*, *M. sharpa*, *M. amplimacula, P. sexmaculata* were 78.2%, 79.6%, 77.4%, 77.3%, and 77.1%, respectively. The AT content distribution of the first codon position (1st), second codon position (2nd) and third codon position (3rd) were 73.5%–75.8%, 69.2%–70.2% and 88.6%–92.8%, respectively; and their order was 3rd > 1st > 2nd. Negative AT (−0.172 to −0.147) and positive GC (0.014–0.047) skews were detected in all 13 PCGs of these five mitogenomes (Table 2).

**Figure 2:**
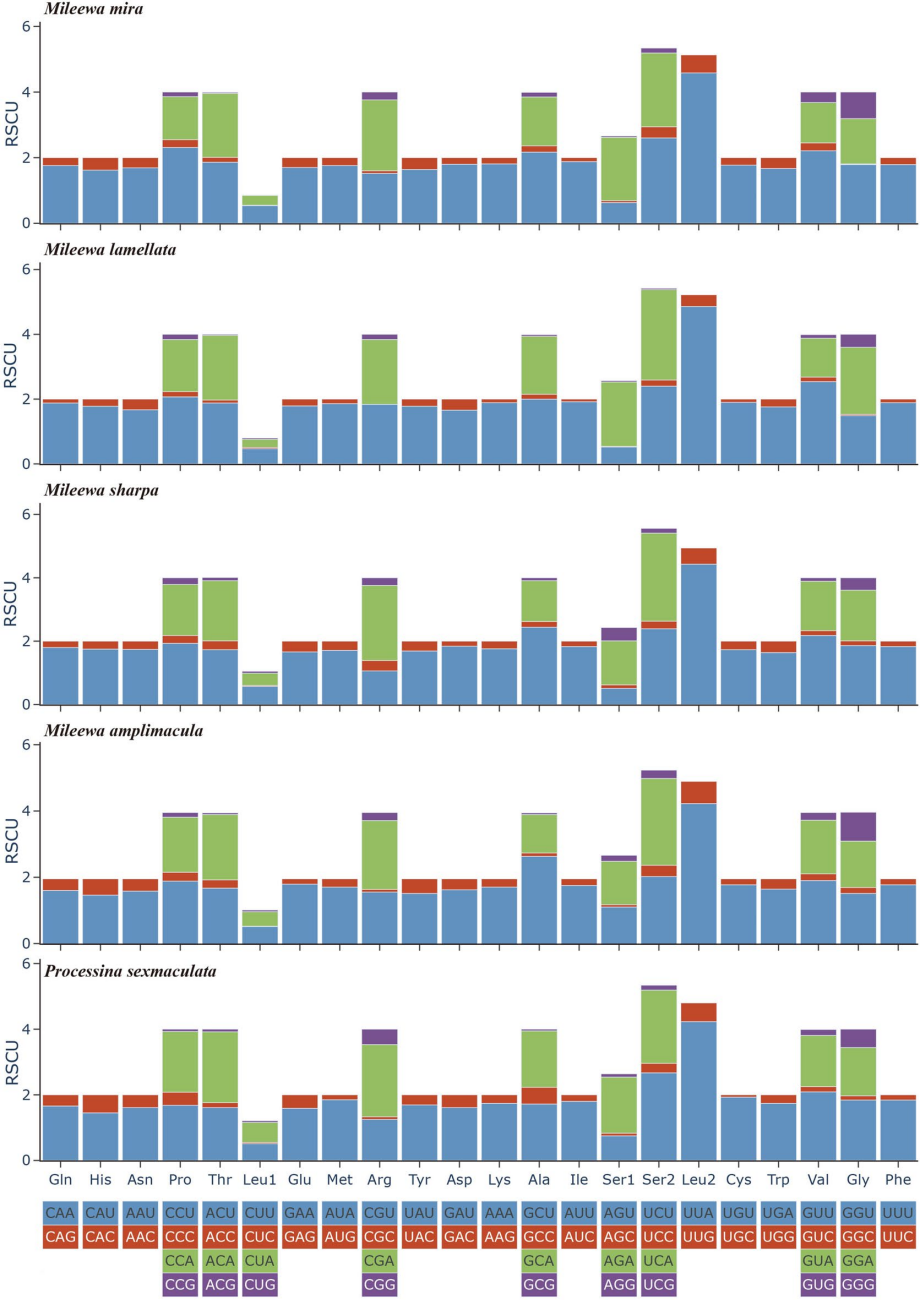
Relative synonymous codon usage (RSCU) values in protein-coding genes (PCGs) of the four *Mileewa* and one *Processina* species mitogenomes.

The relative synonymous codon usage (RSCU) values of the five mitogenomes were shown in Fig. 2 and Table S4. The results indicated that the RSCU values and codon numbers of each mitogenome were similar. The four most frequently used codons were UUU (Phe), UUA (Leu2), AUU (Ile), and AUA (Met), wherein all of which are formed by A and U. This suggests that the most frequently used codons could be found with a preference toward more A and T than G and C.

### Transfer and ribosomal RNA genes

All five new species contained 22 typical tRNA genes; of them, 14 were located on the H-strand, and the other eight on the L-strand (Table 1, Fig. 1). The length of tRNAs ranged 61–72 bp, and the concatenated total length ranged from 1422 bp (*M. sharpa*) to 1440 bp (*M. amplimacula*). The tRNA genes also presented a high AT bias with AT content ranging from 79.9% to 81.4%, and all exhibited a positive AT skew (0.014–0.02, except for −0.003 in *M. amplimacula*) and GC skew (0.16–0.197) (Table 2). The secondary structures of the 22 tRNAs in the five newly sequenced mitogenomes were generated and are presented in Figure S1–S5. All tRNAs could be folded into the typical cloverleaf secondary structure, except for trnS1, which lacks the dihydrouridine (DHU) arm and was replaced by a simple loop, which has been observed in other Cicadellidae mitogenomes^18,42,43,45–47^. In addition, anticodons of all tRNAs were identified across the reported Cicadellidae species^18,48,49^. Several mismatched base pairs, for instance, UU, UG, and an extra A nucleotide, were detected among all tRNAs of the five species. Some single A mismatches appeared in both the trnS1 (anticodon arms) and trnR (acceptor arms) genes within the five newly sequenced mitogenomes (Figure S1–S5). The full size of the two rRNA genes among them ranged from 1920 bp (*M. sharpa*) to 1974 bp (*M. amplimacula*), with a higher AT content (80.3%–81.3%) than tRNA genes. Both l-rRNA and s-rRNA were located on the L-strand, and showed a negative AT skew (−0.179 to −0.101) and a positive GC skew (0.196–0.286) (Table 2, Fig. 1). The l-rRNA genes were located between trnL1 and trnV, with sizes ranging from 1180 bp (*M. sharpa*) to 1217 bp (*M. amplimacula*). The s-rRNA genes were located between trnV and the CR, with sizes ranging from 740 bp (*M. sharpa*) to 757 bp (*M. amplimacula*).

### Overlapping and intergenic spacers

All overlaps and intergenic spacers were identified and were displayed in Table 1. We identified 10 (*P. sexmaculata*) to 16 (*M. sharpa*) overlaps distributed in five mitogenomes ranged from 1 to 12 bp. The longest overlap was 12 bp in *M. mira* and located between nad6 and cytb. The five mitogenomes had 5–14 intergenic spacers, with sizes ranging from 1 to 24 bp. The longest (24 bp) intergenic spacer was identified between the trnS2 and cytb genes in *P. sexmaculata*. Two and three locations had same intergenic spacers and overlaps between genes across the five mitogenomes, respectively: trnP-nad6 and nad4L-trnT (2-bp long intergenic spacers), trnN-trnS1 (1-bp long overlaps), atp6-atp8 (7-bp long overlaps), and trnW-trnC (8-bp long overlaps). Additionally, the differences in overlaps and intergenic spacers between the four *Mileewa* mitogenomes and *P. sexmaculata* mitogenome were found to be located at nad4-nad4L (7-bp long overlaps in four *Mileewa* species; 1-bp long in *P. sexmaculata*) and nad5-trnF (1-bp long overlaps in four *Mileewa* species; 3-bp long intergenic spacers in *P. sexmaculata*). Furthermore, 23-bp (nad4-trnH), 13-bp (cox1-trnL2), and 14-bp long (trnY-cox1) intergenic spacers were identified in the mitogenome of *P. sexmaculata* but not in any of the other Mileewinae species.

### Control region

The CR in the five newly sequenced Mileewinae mitogenomes was located between s-rRNA and trnI, with variable sizes ranging from 446 bp (*M. lamellata*) to 1062 bp (*M. amplimacula*) (Fig. 1, Table 1). The CR was the longest non-coding region, with the highest AT content, ranging from 83.3% (*P. sexmaculata*) to 86.6% (*M. amplimacula*). Except for −0.059 in *P. sexmaculata*, AT skew was positive (0.004–0.057). In addition, *M. lamellata* (0.096) and *P. sexmaculata* (0.042) had a positive GC skew in the CR, whereas the remaining three species had a negative GC skew (−0.06 to −0.03) (Table 2). The detailed structural information of the CR among the five mitogenomes is shown in Figure S6–S10. The number of repeat units in the CR across the five mitogenomes were as follows: one repeat unit in *M. sharpa* (2 × 107 bp) and *M. mira* (2 × 168 bp), two in *M. lamellata* (unit 1, 2 × 15 bp; unit 2, 2 × 79 bp), three in *M. amplimacula* (unit 1, 2 × 39 bp; unit 2, 2 × 178 bp; unit 3, 2 × 144 bp), and more abundant units in *P. sexmaculata* (unit 1, 3 × 41 bp; unit 2, 2 × 151 bp; unit 3, 4 × 47 bp; unit 4, 2 × 102 bp). The largest repeat unit was 178-bp long in *M. amplimacula,* whereas the smallest with a size of 15 bp was found in *M. lamellata*; they were both contained two repeats.

### Phylogenetic relationships

**Figure 3:**
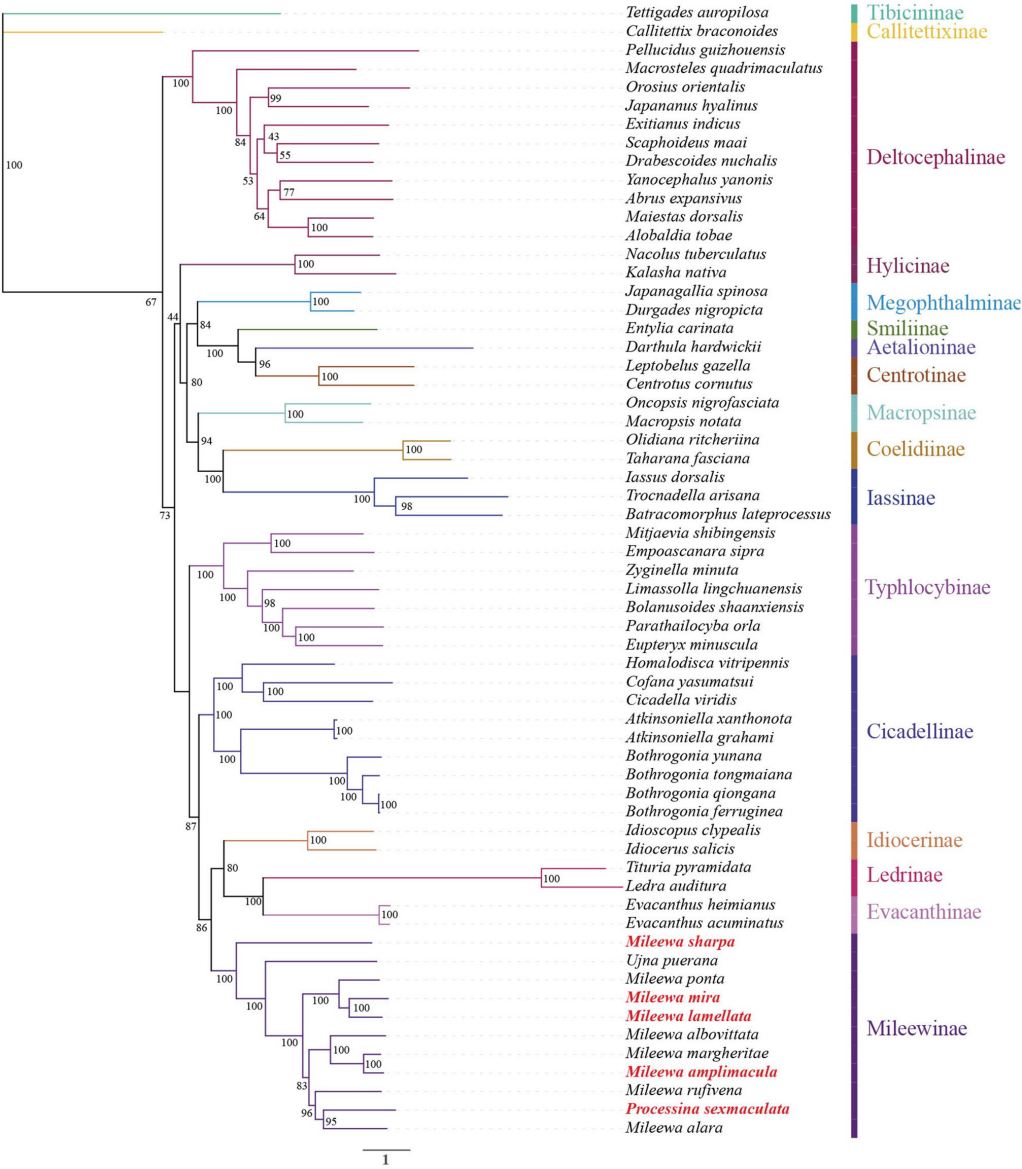
Maximum likelihood (ML) phylogenetic tree analysis of 13 protein-coding genes (PCGs). *Callitettix braconoides* and *Tettigades auropilosa* are outgroups. Bootstrap support (BS) values are shown at the nodes. Newly sequenced mitogenomes are indicated in red.

**Figure 4:**
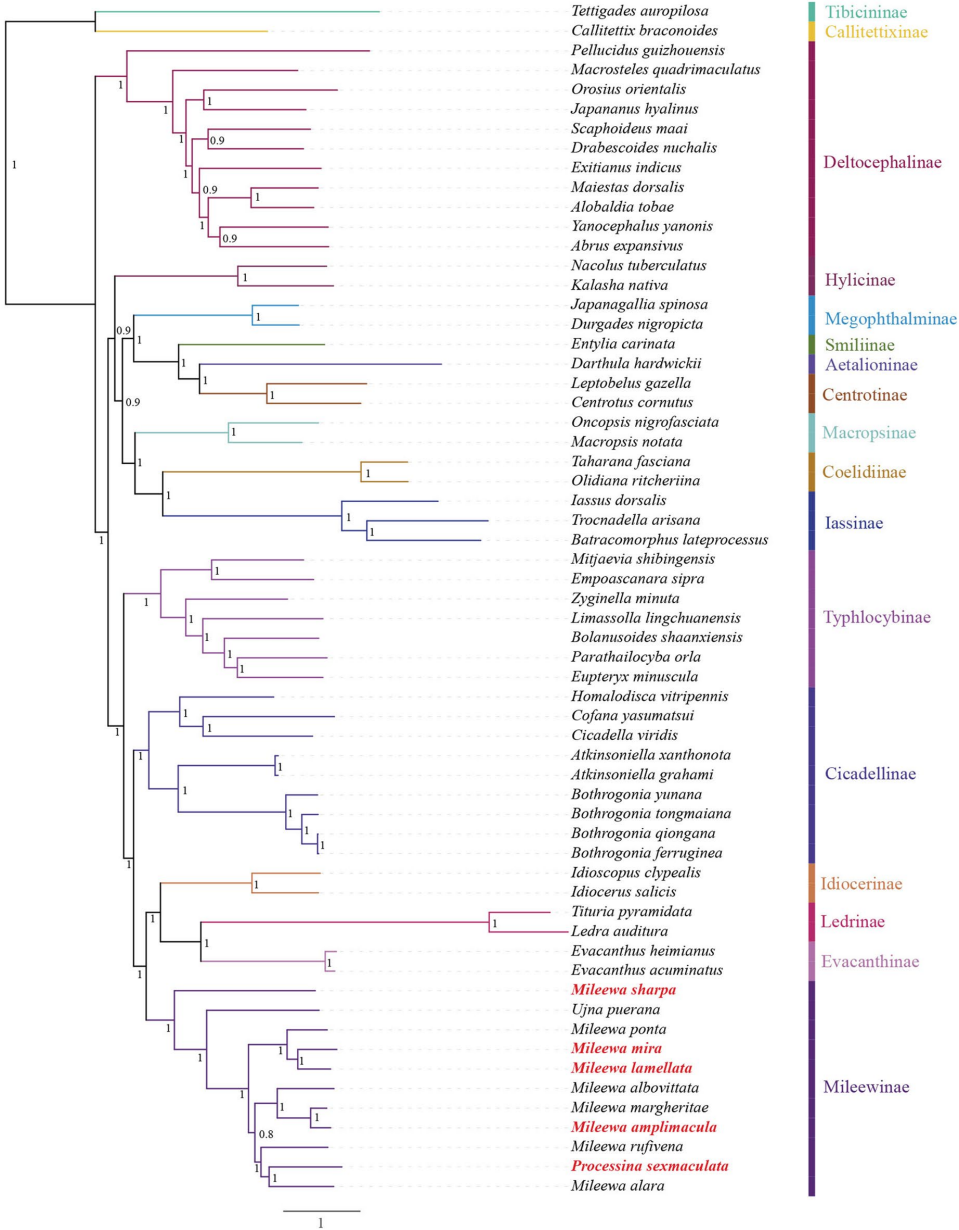
Bayesian inference (BI) phylogenetic tree analysis of 13 protein-coding genes (PCGs). *Callitettix braconoides* and *Tettigades auropilosa* are outgroups. Posterior probability (PP) values are shown at the nodes. Newly sequenced mitogenomes are indicated in red.

Phylogenetic relationships were analyzed using 13 PCGs of 59 Membracoidea species and two outgroups mitogenome datasets. Two phylogenetic trees were reconstructed using the BI and ML methods (BI-PCGS and ML-PCGS, respectively) (Figs. 3,4). According to these two trees, 11 Mileewinae species formed a monophyletic group with high support values (BS = 100, PP = 1) and were located at the apex of the trees, and each subfamily was completely recovered as a monophyletic group in both ML and BI analyses. Both analyses demonstrated that 11 Mileewinae species were clustered together with a sister relationship with the subfamilies Idiocerinae, Evacanthinae, and Ledrinae, and these four subfamilies (Mileewinae, Idiocerinae, Evacanthinae, and Ledrinae) clustered together to form a sister group to Cicadellinae, with high support values (BS > 86, PP = 1). Moreover, the subfamilies Mileewinae, Idiocerinae, Evacanthinae, Ledrinae, and Cicadellinae clustered in one clade to form a sister group to Typhlocybinae. Our study indicated that Typhlocybinae is more ancient than Mileewinae and Cicadellinae and that Mileewinae is not a sister group of Typhlocybinae, which differs from a previous study^18,42,45,50^.

## Discussion

In the present study, we sequenced and comparatively analyzed the complete mitogenomes of five species belonging to two genera of Mileewinae. *Processina sexmaculata* was the first sequenced species from the genus *Processina* Yang, Deitz & Li, 2005, which was established in China. The length of the mitogenomes of the five Mileewinae species ranged from 14787 bp (*M. lamellate)* to 15436 bp (*M. amplimacula*). We compared these five mitogenomes to those of established Mileewinae species^18–22^ and found that the length of each PCG and tRNA gene is quite similar, while the difference is mainly observed in rRNAs and CRs. All the PCGs have relatively conserved characteristics. We found, for the first time, that a single A mismatch appeared in both the trnS1 (anticodon arms) and trnR (acceptor arms) genes in the five newly sequenced mitogenomes. Some common overlapping and intergenic spacer regions across the five mitogenomes were identified as well, such as a 7-bp long overlapping region between atp8 and atp6, an 8-bp long overlap between trnC and trnW, and a 2-bp long intergenic spacer region present in both trnP-nad6 and trnT-nad4L. Moreover, we found that *P. sexmaculata*, which is the first *Processina* species with a sequenced mitogenome, has a 23-bp long intergenic spacer located between trnH and nad4. A 24-bp intergenic spacer was found between cytb and trnS2 in *P. sexmaculata* mitogenome but not in the mitogenomes of other Mileewinae species.

We further generated the BI and ML trees with concatenated alignment of PCGs. Our results indicated that each subfamily well separated, and their monophyly weresupported. The phylogenetic relationships were as follows: (Deltocephalinae + ((Hylicinae + ((Megophthalminae + (Smiliinae + (Aetalioninae + Centrotinae))) + (Macropsinae + (Coelidiinae + Iassinae)))) + (Typhlocybinae + (Cicadellinae + (Mileewinae + (Idiocerinae + (Evacanthinae + Ledrinae)))))) (Figs. 3,4). The BI-PCGS tree had more higher support values than the ML-PCGS tree; this topology is not completely consistent with the findings of recent studies^18,42,45,51^, possibly due to the difference of the data and phylogeny models used. Furthermore, 11 Mileewinae species clustered in a monophyletic clade (PP = 1, BS = 100), which is consistent with the studies by Yu et al., Dietrich et al., and our previous study^18–22,52^. We also found that Mileewinae was at the apex of the phylogenetic tree, while Deltocephalinae appeared at the basal branch of the trees, which was partly the same result as that reported by Yu et al. In addition, Cicadellinae, Typhlocybinae, and Mileewinae were each recovered as a monophyletic group. In contrast to the studies by Yu et al. and Dietrich et al.^18,50^, based on the phylogenetic trees from this study (Figs. 3, 4), we considered that Typhlocybinae is more ancient than Mileewinae and Cicadellinae and suggested that Typhlocybinae is not a sister group to Mileewinae. Within the Mileewinae subfamily, all species maintained the same relationships and topologies according to both the BI and ML analyses. The phylogenetic relationships with high support values (PP > 0.8, BS > 83) within the subfamily Mileewinae were as follows: (*M. sharpa* + (*U. puerana* + ((*M. ponta* + (*M. mira* + *M. lamellata*)) + ((*M. albovittata* + (*M. margheritae* + *M. amplimacula*)) + (*M. rufivena* + (*P. sexmaculata* + *M. alara*)))))) (Figs. 3,4). These results are also different from those presented in a recent study of Yu et al., who mentioned the topology as (*U. puerana* + (*M. ponta* + (*M. rufivena* + *M. alara*) + (*M. albovittata* + *M. margheritae*))). Among the studied topologies, the monophyly of the genera *Processina, Mileewa* and *Ujna* were not supported. These three genera species of Mileewinae were not clearly separated. Nevertheless, we only used one species from *Processina* that raises concerns about its representativeness. This study provides a referenceable framework to understand the relationships between Mileewinae and other subfamilies and further enriches the mitogenome database of the tribe Mileewini. Molecular data of more variable genera of Mileewinae are required to determine the phylogenetic relationships within this group and define the monophyly of Mileewinae.

## Supplementary Information

Supplementary Information.

## Data Availability

The datasets generated and analyzed during the current study are available in the [NCBI] repository, [accession number: ON464171–ON464175; weblink: https://www.ncbi.nlm.nih.gov/nuccore/2314113360, https://www.ncbi.nlm.nih.gov/nuccore/2314113374, https://www.ncbi.nlm.nih.gov/nuccore/2314113388, https://www.ncbi.nlm.nih.gov/nuccore/2314113402, https://www.ncbi.nlm.nih.gov/nuccore/2314113416].

## References

[1] Evans JW (1947). A natural classification of leaf-hoppers (Jassoidea, Homoptera) part 3: Jassidae. Trans. R. Entomol. Soc. Lond..

[2] Young DA (1965). Western hemisphere mileewanini (Homoptera, Cicadellidae). Zool. Beitr..

[3] Mahmood SH (1967). A study of the typhlocybine genera of the oriental region. Pac. Insects Monogr..

[4] Young DA (1968). Taxonomic study of the cicadellinae (Homoptera: Cicadellidae) Pt. 1: Proconiini. Bull. U. S. Natl Mus.

[5] Dietrich, C.; Dmitriev, D.; Rakitov, R.; Takiya, D.; Zahniser, J. Phylogeny of Cicadellidae (Cicadomorpha: Membracoidea) Based on Combined Morphological and 28s rDNA Sequence Data. In* Purcell A (Ed) Abstracts of Talks and Posters: 12th International Auchenorrhyncha Congress, Berkeley, CA, 7–12*, S713–14 (2005).

[6] Dietrich C (2011). Tungurahualini, a new tribe of neotropical leafhoppers, with notes on the subfamily mileewinae (Hemiptera, Cicadellidae). ZooKeys.

[7] Krishnankutty S, Dietrich C (2011). Review of mileewine leafhoppers (Hemiptera: Cicadellidae: Mileewinae) in madagascar, with description of seven new species. Ann. Entomol. Soc. Am..

[8] Distant, W. The Fauna of British India, Including Ceylon and Burma. Rhynchota 4 1908, Part 1–2, 156–419.

[9] Yang M-F, Deitz L, Li Z-Z (2005). A new genus and two new species of cicadellinae from China (Hemiptera: Cicadellidae), with a key to the Chinese genera of cicadellinae. J. N. Y. Entomol. Soc..

[10] Yan B, He H-L, Yang M-F, Webb M (2021). A new genus and species of mileewini leafhoppers (Hemiptera, Cicadellidae, Mileewinae) from China, with a key to genera. ZooKeys.

[11] He H-L, Yan B, Yang M-F, Webb M (2021). Four new species of mileewini leafhoppers (Hemiptera: Cicadellidae: Mileewinae) from China, with a checklist to Chinese species. Zootaxa.

[12] He H-L, Yan B, Yang M-F (2021). Two new species of the leafhopper tribe mileewini (Hemiptera: Cicadellidae: Mileewinae) from Hainan Island, China, with a key to species. Zootaxa.

[13] Yu X-F, He H-L, Yang M-F (2021). Three new species of *Mileewa* (Hemiptera: Cicadellidae: Mileewinae) from tibet China. Zootaxa.

[14] Yang, M.-F.; Meng, Z.-H.; Li, Z.-Z. *Hemiptera: Cicadellidae (II): Cicadellinae Fauna Sinica: Insecta, 67 Beijing*; pp. 1–637 (2017).

[15] Boore JL (1999). Animal mitochondrial genomes. Nucleic Acids Res..

[16] Cameron SL (2014). Insect mitochondrial genomics: Implications for evolution and phylogeny. Annu. Rev. Entomol..

[17] Wolstenholme DR (1992). Animal mitochondrial DNA: Structure and evolution. Int. Rev. Cytol..

[18] Yu T, Zhang Y-L (2021). Two complete mitochondrial genomes of mileewinae (Hemiptera: Cicadellidae) and a phylogenetic analysis. Insects.

[19] He H-L, Yang M-F (2020). Characterization and phylogenetic analysis of the mitochondrial genome of *Mileewa ponta* (Hemiptera: Cicadellidae: Mileewinae). Mitochondrial DNA Part B.

[20] He H-L, Yang M-F (2020). The mitogenome of *Mileewa margheritae* (Hemiptera: Cicadellidae: Mileewinae). Mitochondrial DNA Part B.

[21] He H-L, Li H-X, Yang M-F (2019). Complete mitochondrial genome sequence of *Mileewa albovittata* (Hemiptera: Cicadellidae: Mileewinae). Mitochondrial DNA Part B.

[22] He H-L, Yang M-F (2021). Characterization of the leafhopper mitogenome of *Mileewa alara* (Hemiptera: Cicadellidae: Mileewinae) and Its phylogenetic analysis. Mitochondrial DNA Part B.

[23] Jin J-J, Yu W-B, Yang J-B, Song Y, DePamphilis CW, Yi T-S, Li D-Z (2020). GetOrganelle: A fast and versatile toolkit for accurate de novo assembly of organelle genomes. Genome Biol..

[24] Meng G, Li Y, Yang C, Liu S (2019). MitoZ: A toolkit for animal mitochondrial genome assembly annotation and visualization. Nucleic Acids Res..

[25] Bernt M, Donath A, Jühling F, Externbrink F, Florentz C, Fritzsch G, Pütz J, Middendorf M, Stadler PF (2013). MITOS: Improved de novo metazoan mitochondrial genome annotation. Mol. Phylogenet. Evol..

[26] Laslett D, Canbäck B (2008). ARWEN: A program to detect tRNA genes in metazoan mitochondrial nucleotide sequences. Bioinformatics.

[27] Greiner S, Lehwark P, Bock R (2019). OrganellarGenomeDRAW (OGDRAW) version 1.3.1: Expanded toolkit for the graphical visualization of organellar genomes. Nucleic Acids Res..

[28] Zhang D, Gao F, Jakovlić I, Zou H, Zhang J, Li WX, Wang GT (2020). PhyloSuite: An integrated and scalable desktop platform for streamlined molecular sequence data management and evolutionary phylogenetics studies. Mol. Ecol. Resour..

[29] Perna NT, Kocher TD (1995). Patterns of nucleotide composition at fourfold degenerate sites of animal mitochondrial genomes. J. Mol. Evol..

[30] Benson G (1999). Tandem repeats finder: A program to analyze DNA sequences. Nucleic Acids Res..

[31] Lehwark P, Greiner S (2019). GB2sequin-a file converter preparing custom genbank files for database submission. Genomics.

[32] Liu J, Bu C, Wipfler B, Liang A (2014). Comparative analysis of the mitochondrial genomes of callitettixini spittlebugs (Hemiptera: Cercopidae) confirms the overall high evolutionary speed of the AT-rich region but reveals the presence of short conservative elements at the tribal level. PLoS ONE.

[33] Katoh K, Standley DM (2013). MAFFT multiple sequence alignment software version 7: Improvements in performance and usability. Mol. Biol. Evol..

[34] Ranwez V, Douzery EJ, Cambon C, Chantret N, Delsuc F (2018). MACSE V2: Toolkit for the alignment of coding sequences accounting for frameshifts and stop codons. Mol. Biol. Evol..

[35] Talavera G, Castresana J (2007). Improvement of phylogenies after removing divergent and ambiguously aligned blocks from protein sequence alignments. Syst. Biol..

[36] Lanfear R, Frandsen PB, Wright AM, Senfeld T, Calcott B (2017). PartitionFinder 2: New methods for selecting partitioned models of evolution for molecular and morphological phylogenetic analyses. Mol. Biol. Evol..

[37] Lanfear R, Calcott B, Ho SY, Guindon S (2012). PartitionFinder: Combined selection of partitioning schemes and substitution models for phylogenetic analyses. Mol. Biol. Evol..

[38] Nguyen L-T, Schmidt HA, Von Haeseler A, Minh BQ (2015). IQ-TREE: A fast and effective stochastic algorithm for estimating maximum-likelihood phylogenies. Mol. Biol. Evol..

[39] Ronquist F, Teslenko M, Van Der Mark P, Ayres DL, Darling A, Höhna S, Larget B, Liu L, Suchard MA, Huelsenbeck JP (2012). MrBayes 3.2: Efficient bayesian phylogenetic inference and model choice across a large model space. Syst. Biol..

[40] Minh BQ, Nguyen MAT, von Haeseler A (2013). Ultrafast approximation for phylogenetic bootstrap. Mol. Biol. Evol..

[41] Letunic I, Bork P (2021). Interactive tree of life (iTOL) v5: An online tool for phylogenetic tree display and annotation. Nucleic Acids Res..

[42] Jiang Y, Li H-X, Yu X-F, Yang M-F (2021). Characterization of two complete mitochondrial genomes of *Atkinsoniella* (Hemiptera: Cicadellidae: Cicadellinae) and the phylogenetic implications. Insects.

[43] Jiang Y, Li H-X, Yu X-F, Yang M-F (2022). comparative analysis of mitochondrial genomes among twelve sibling species of the genus *Atkinsoniella* distant, 1908 (Hemiptera: Cicadellidae: Cicadellinae) and phylogenetic analysis. Insects.

[44] Ojala D, Montoya J, Attardi G (1981). tRNA punctuation model of RNA processing in human mitochondria. Nature.

[45] Wang X, Wang J, Dai R (2021). Structural features of the mitogenome of the leafhopper genus *Cladolidia* (Hemiptera: Cicadellidae: Coelidiinae) and phylogenetic implications in cicadellidae. Ecol. Evol..

[46] Lin S, Huang M, Zhang Y (2021). Structural features and phylogenetic implications of 11 new mitogenomes of typhlocybinae (Hemiptera: Cicadellidae). Insects.

[47] Wang J, Wu Y, Dai R, Yang M (2020). comparative mitogenomes of six species in the subfamily iassinae (Hemiptera: Cicadellidae) and phylogenetic analysis. Int. J. Biol. Macromol..

[48] Du Y, Dietrich CH, Dai W (2019). Complete mitochondrial genome of *Macrosteles quadrimaculatus* (Matsumura)(Hemiptera: Cicadellidae: Deltocephalinae) with a shared tRNA rearrangement and its phylogenetic implications. Int. J. Biol. Macromol..

[49] Du Y, Liang Z, Dietrich CH, Dai W (2021). comparative analysis of mitochondrial genomes of nirvanini and evacanthini (Hemiptera: Cicadellidae) reveals an explicit evolutionary relationship. Genomics.

[50] Dietrich, C.H. The Role of Grasslands in the Diversification of Leafhoppers (Homoptera: Cicadellidae): A Phylogenetic Perspective. In *Proceedings of the Proceedings of the fifteenth north american prairie conference*; Natural Areas Assoc Bend, OR, pp. 44–49 (1999).

[51] Chen X, Yuan Z, Li C, Dietrich CH, Song Y (2021). Structural features and phylogenetic implications of cicadellidae subfamily and two new mitogenomes leafhoppers. PLoS ONE.

[52] Dietrich C, Rakitov R, Holmes J, Black W (2001). Phylogeny of the major lineages of membracoidea (Insecta: Hemiptera: Cicadomorpha) based on 28s rDNA sequences. Mol. Phylogenet. Evol..

