# Author Correction: Characterization and phylogenetic implications of newly sequenced mitogenomes of Five *Mileewa* and *Processina* species from China (Hemiptera: Cicadellidae: Mileewinae)

**DOI:** 10.1038/s41598-023-28116-y

**Published:** 2023-01-17

**Authors:** Hongli He, Bin Yan, Xiaofei Yu, Maofa Yang

**Affiliations:** 1 Institute of Entomology of Guizhou University, Guizhou Provincial Key Laboratory for Agricultural Pest Management of the Mountainous Region, Guiyang, 550025 Guizhou China; 2 College of Tobacco Sciences of Guizhou University, Guiyang, 550025 Guizhou China

Correction to: *Scientific Reports* https://doi.org/10.1038/s41598-022-25376-y, published online 02 December 2022

The Acknowledgments section in the original version of this Article was omitted. The Acknowledgments section now reads:

“We want to thank Xiaoli Xu, Likun Zhong and Yan Jiang for collecting the specimen of this study. This work was funded by Guizhou Province Science and Technology Innovation Talent Team Project (Qian Ke He Pingtai Rencai—CXTD [2021]004).”

The original Article has been corrected.

