# Supplementary material for: Characterization and phylogenetic implications of newly sequenced mitogenomes of Five Mileewa and Processina species from China (Hemiptera: Cicadellidae: Mileewinae)
Source: Sci Rep. 2022 Dec 2;12:20852. doi: 10.1038/s41598-022-25376-y (PMC9718768; doi:10.1038/s41598-022-25376-y)
Supplement: Supplementary file 1 — Supplementary Information. [file 41598_2022_25376_MOESM1_ESM.docx]

Supplementary Information

**Characterization and Phylogenetic Implications of Newly Sequenced Mitogenomes of Five *Mileewa* and *Processina* Species from China (Hemiptera: Cicadellidae: Mileewinae)**

Hongli He^a^, Bin Yan^a^, Xiaofei Yu^b^, Maofa Yang^a, b^

a Institute of Entomology of Guizhou University, Guizhou Provincial Key Laboratory for Agricultural Pest Management of the Mountainous Region, Guiyang, 550025, Guizhou, China

b College of Tobacco Sciences of Guizhou University, Guiyang, 550025, Guizhou, China

Corresponding author: Maofa Yang. E-mail: gdgdly@126.com


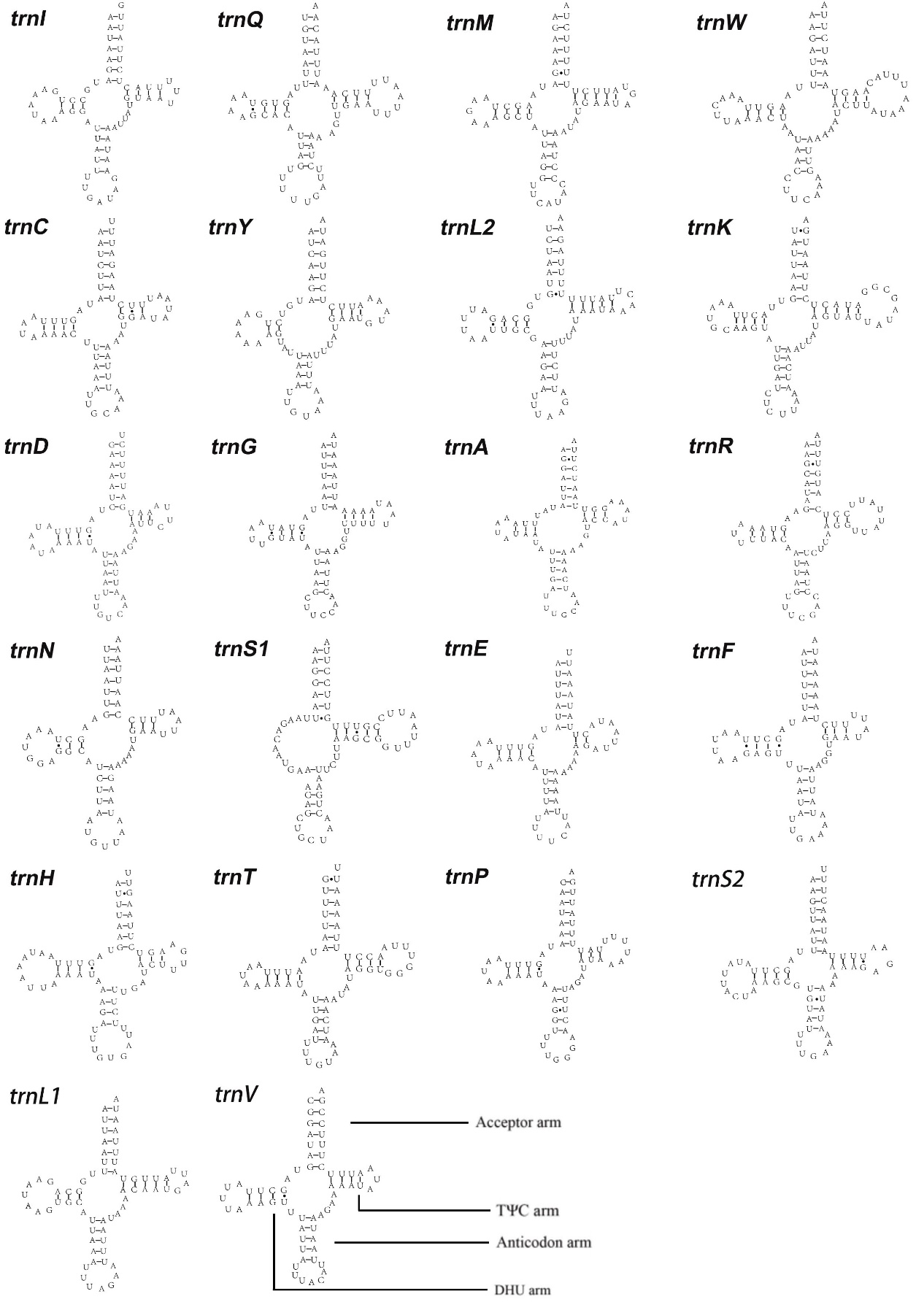


Figure S1. Predicted secondary structures for the 22 transfer RNAs (tRNAs) of the *Mileewa mira* mitogenome. Dashes indicate Watson–Crick base pairing, and dots indicate UG base pairing.


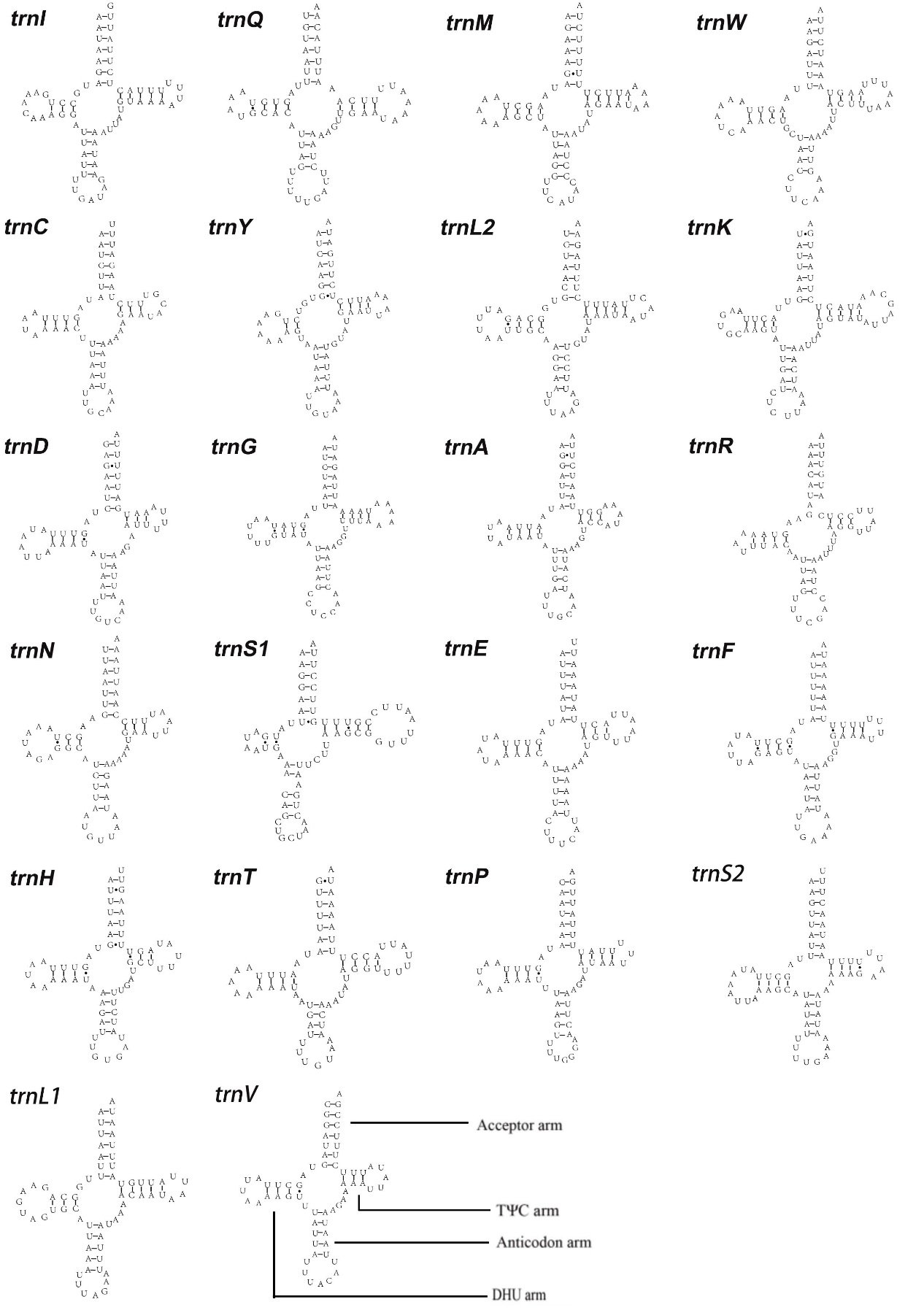


Figure S2. Predicted secondary structures for the 22 transfer RNAs (tRNAs) of the *Mileewa lamellata* mitogenome. Dashes indicate Watson–Crick base pairing, and dots indicate UG base pairing.


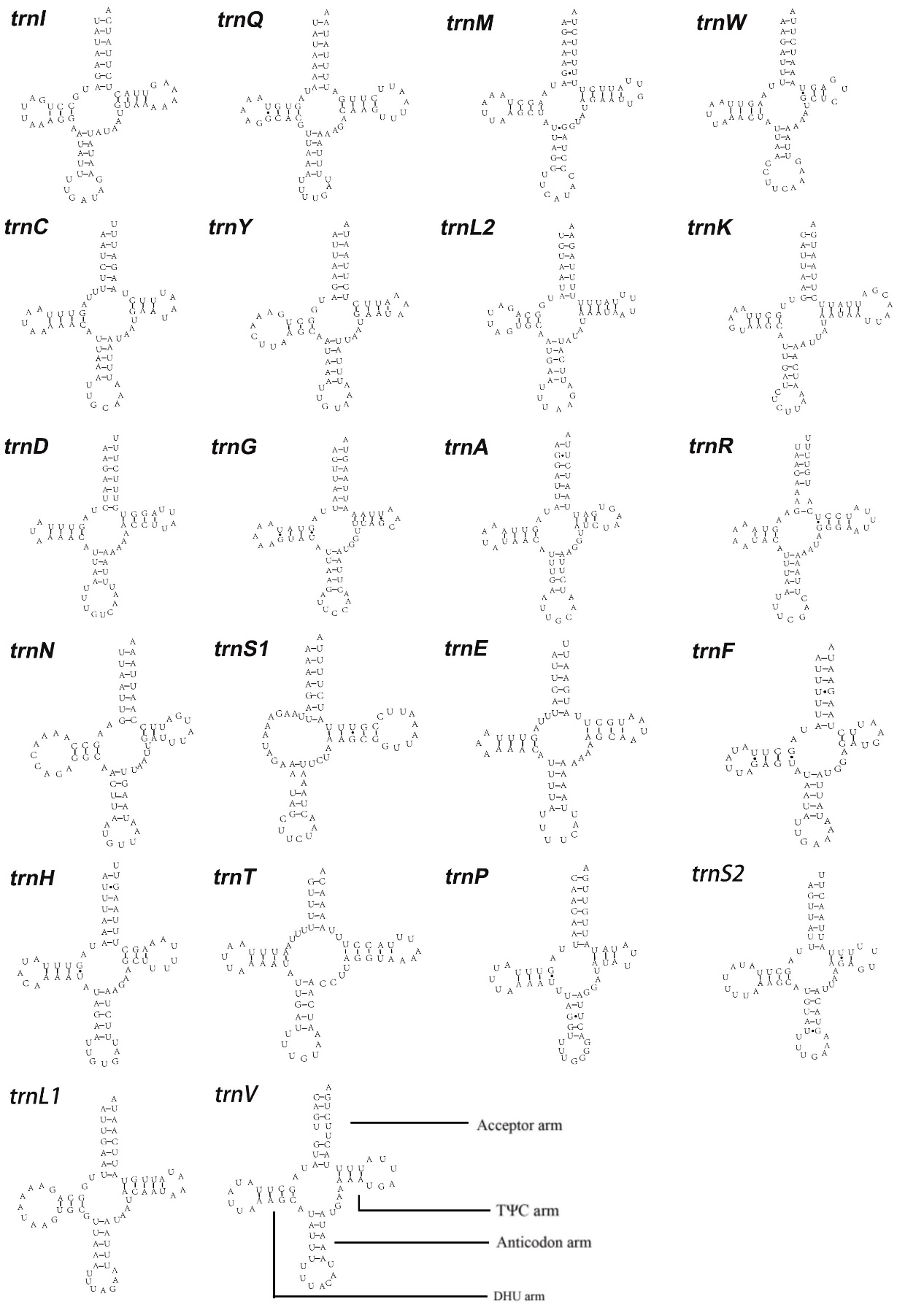


Figure S3. Predicted secondary structures for the 22 transfer RNAs (tRNAs) of the *Mileewa sharpa* mitogenome. Dashes indicate Watson–Crick base pairing, and dots indicate UG base pairing.


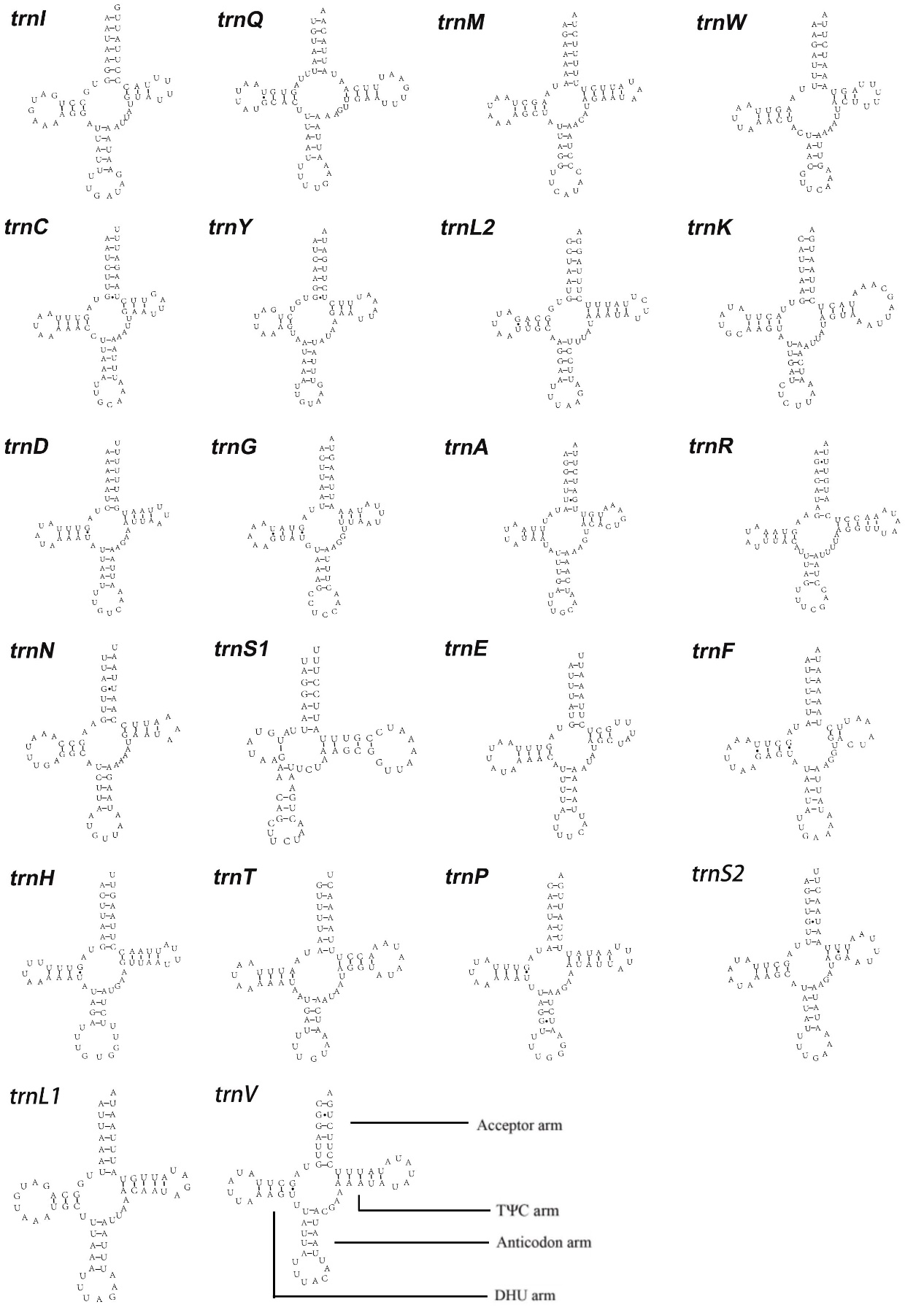


Figure S4. Predicted secondary structures for the 22 transfer RNAs (tRNAs) of the *Mileewa amplimacula* mitogenome. Dashes indicate Watson–Crick base pairing, and dots indicate UG base pairing.


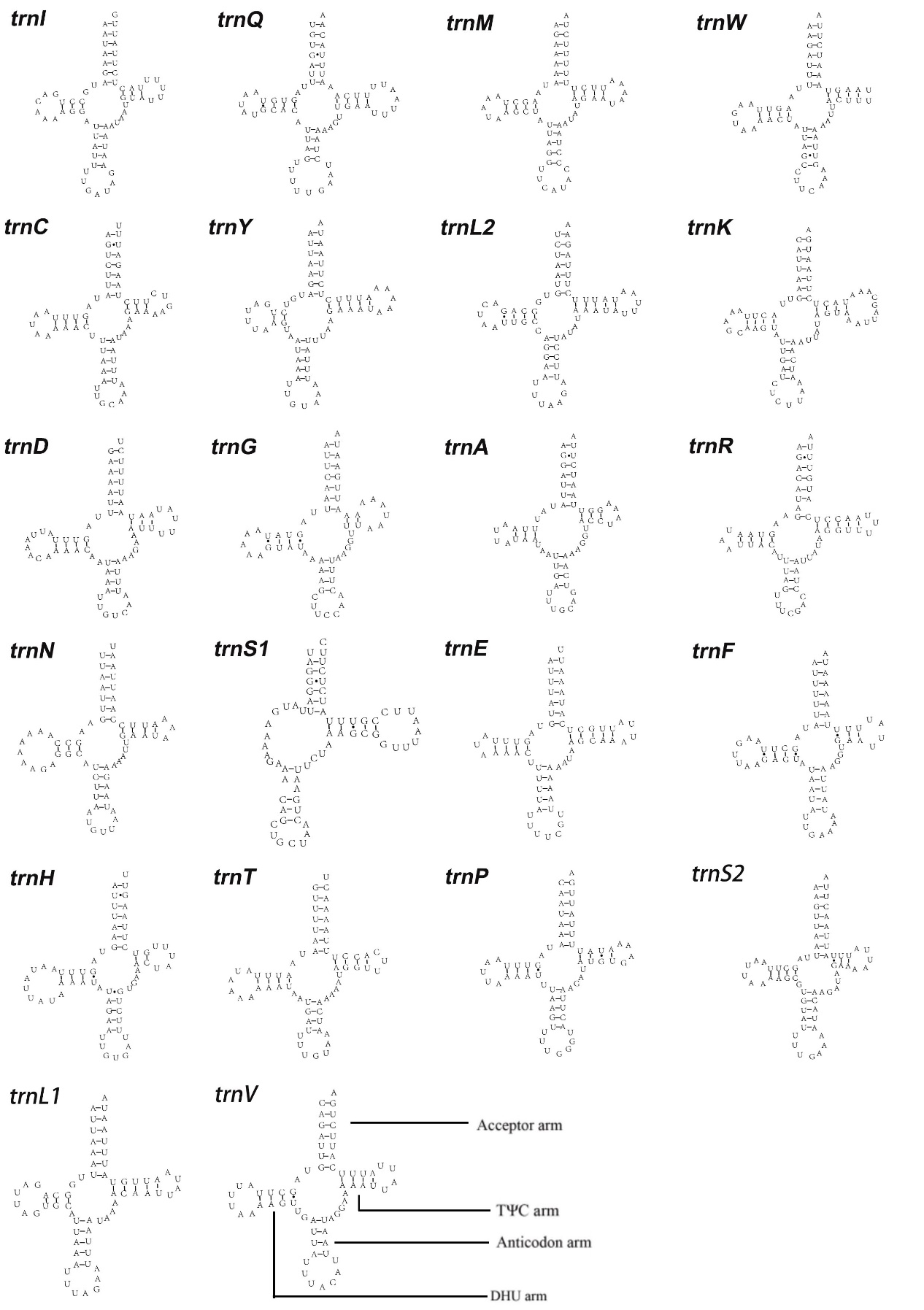


Figure S5. Predicted secondary structures for the 22 transfer RNAs (tRNAs) of the *Processina sexmaculata* mitogenome. Dashes indicate Watson–Crick base pairing, and dots indicate UG base pairing.


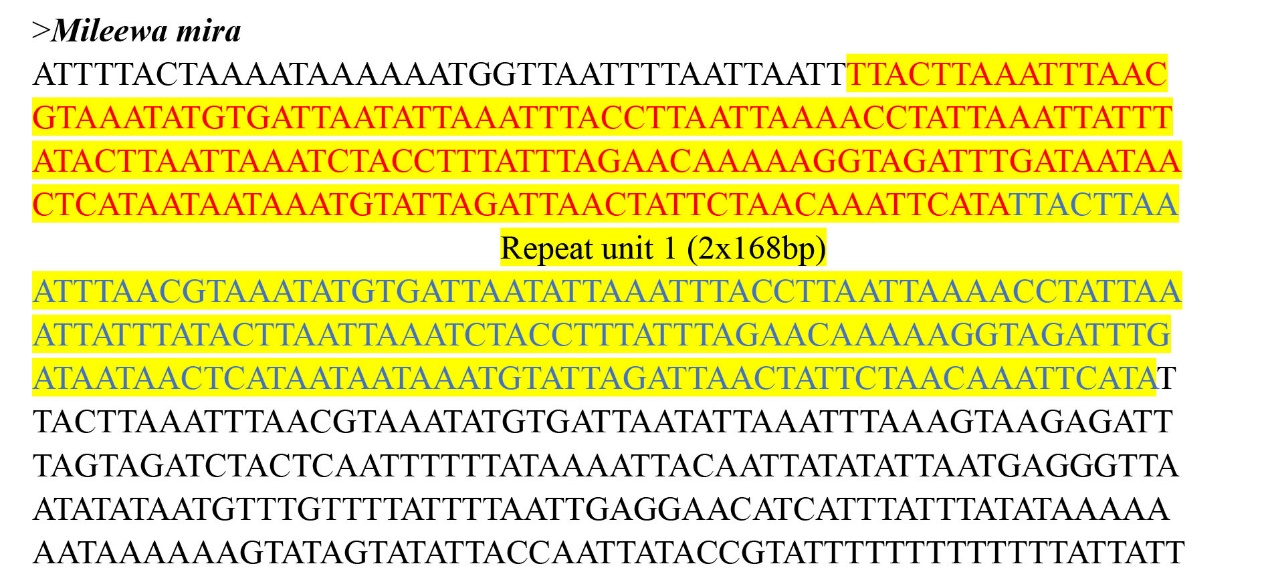


Figure S6. Organization of the control region in *Mileewa mira* mitogenome. Different tandem repeats unit are marked with different colors. * indicates a mismatch; –indicates an insertion or deletion.


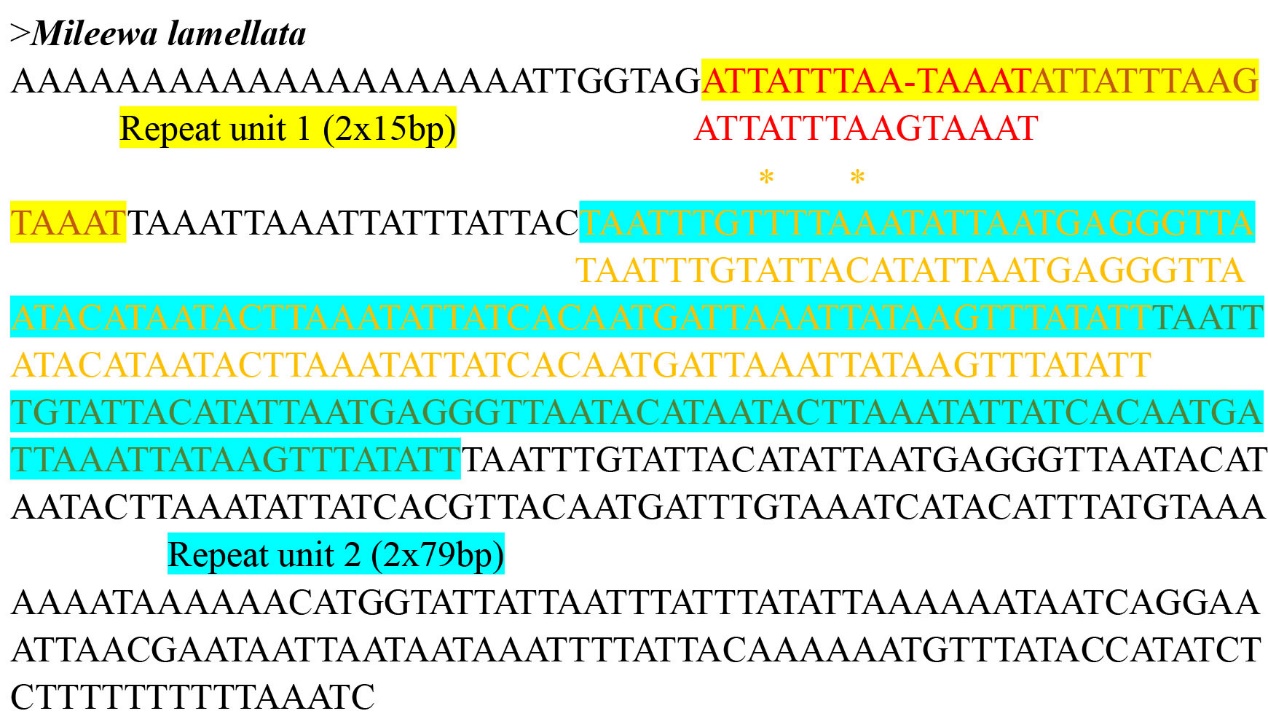


Figure S7. Organization of the control region in *Mileewa lamellata* mitogenome. Different tandem repeats unit are marked with different colors. * indicates a mismatch; –indicates an insertion or deletion.


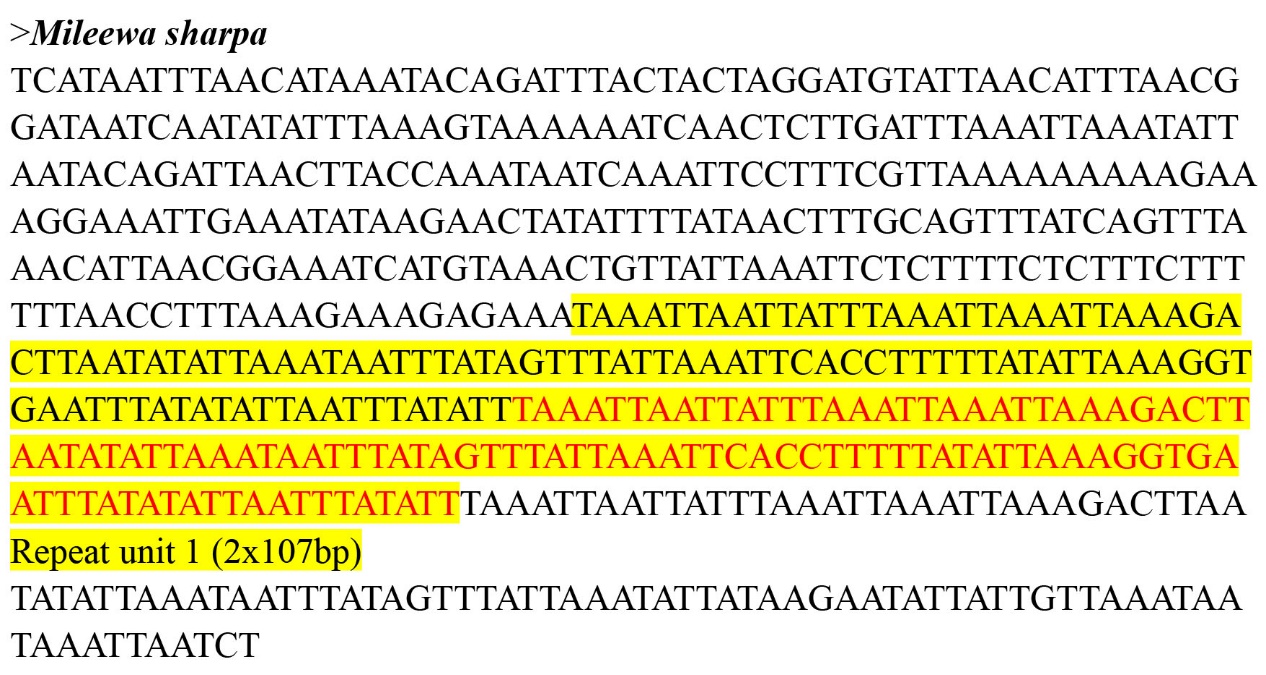


Figure S8. Organization of the control region in *Mileewa sharpa* mitogenome. Different tandem repeats unit are marked with different colors. * indicates a mismatch; –indicates an insertion or deletion.


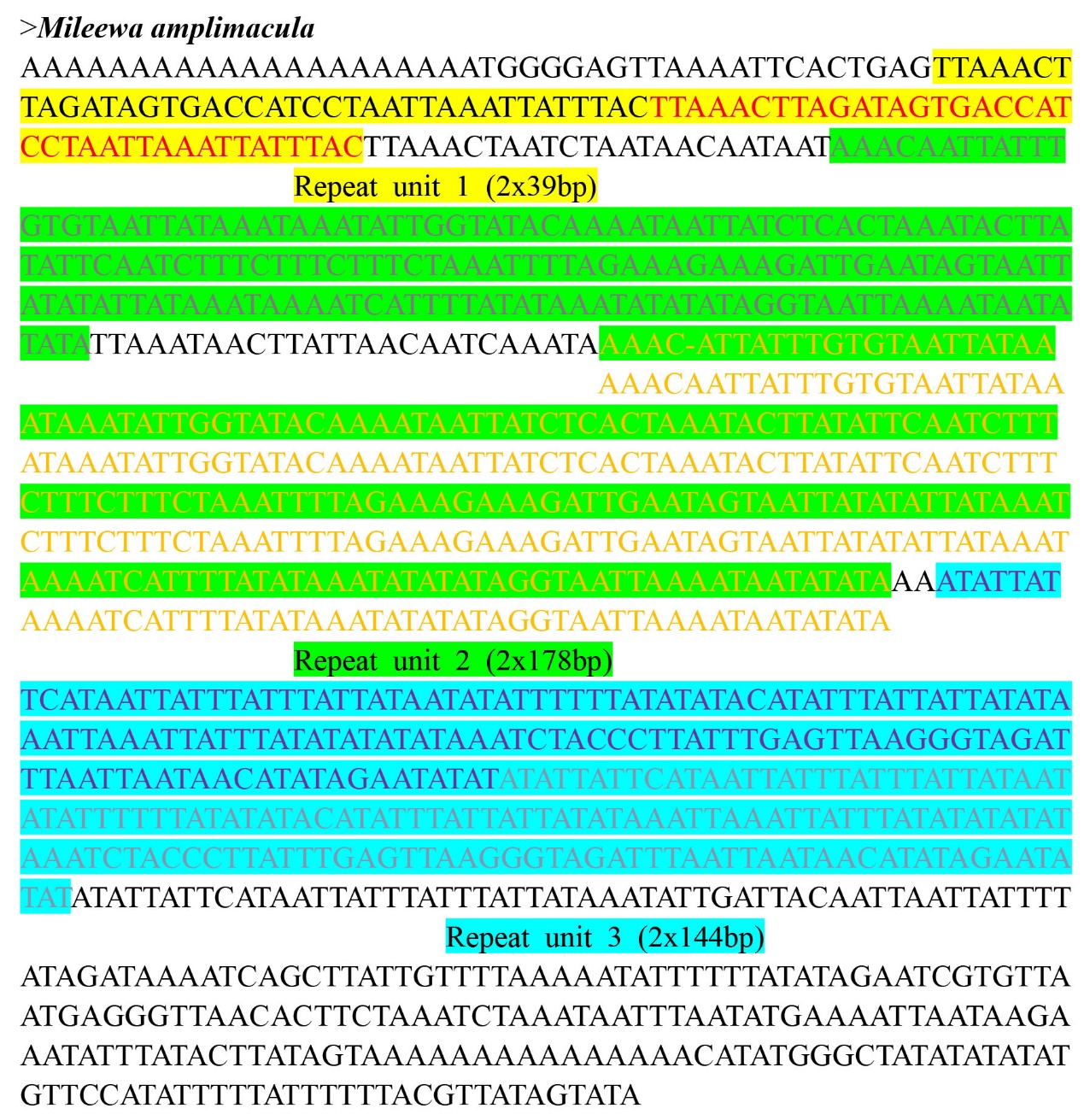


Figure S9. Organization of the control region in *Mileewa amplimacula* mitogenome. Different tandem repeats unit are marked with different colors. * indicates a mismatch; –indicates an insertion or deletion.


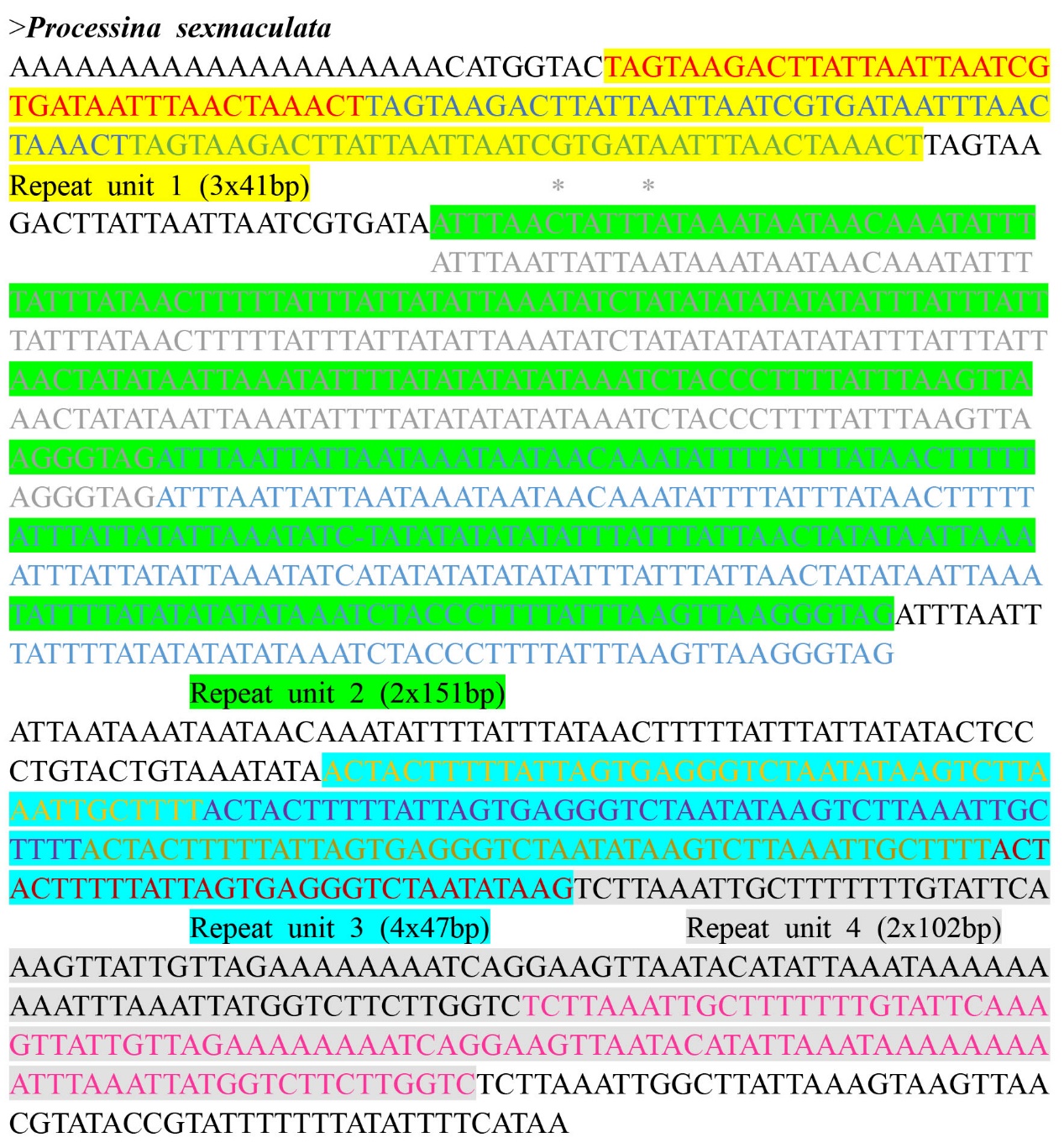


Figure S10. Organization of the control region in *Processina sexmaculata* mitogenome. Different tandem repeats unit are marked with different colors. * indicates a mismatch; –indicates an insertion or deletion.

Table S1. Specimen collection information for five Mileewinae species used in this study.

| Name | Locality | collection time | collector |
| --- | --- | --- | --- |
| *Mileewa mira* | Maolan National Natural Reserve, Guizhou, China | 13-April-2019 | Xiaoli-Xu |
| *Mileewa lamellata* | Yunkai Mountain, Guangdong, China | 25-April-2019 | Yan Jiang |
| *Mileewa sharpa* | Wuzhi Mountain, Hainan, China | 13-June-2019 | Likun-Zhong |
| *Mileewa amplimacula* | Mengyuan town, Yunnan, China | 15-November-2018 | Likun-Zhong |
| *Processina sexmaculata* | Mengla county, Yunnan, China | 16-November-2018 | Likun-Zhong |

Table S2. Mitochondrial genomes used for phylogenetic analyses in this study.

| Superfamily | Family | Subfamily | Species | Accession Number |
| --- | --- | --- | --- | --- |
| Cicadoidea | Cicadidae | Tibicininae | *Tettigades auropilosa* | KM000129 |
| Cercopoidea | Cercopidae | Callitettixinae | *Callitettix braconoides* | NC_025497 |
| Membracoidea | Aetalionidae | Aetalioninae | *Darthula hardwickii* | NC_026699 |
| Membracoidea | Membracidae | Centrotinae | *Centrotus cornutus* | KX437728 |
| Membracoidea | Membracidae | Centrotinae | *Leptobelus gazella* | NC_023219 |
| Membracoidea | Cicadellidae | Cicadellinae | *Bothrogonia ferruginea* | KU167550 |
| Membracoidea | Cicadellidae | Cicadellinae | *Cicadella viridis* | MK335936 |
| Membracoidea | Cicadellidae | Cicadellinae | *Atkinsoniella grahami* | MW533712 |
| Membracoidea | Cicadellidae | Cicadellinae | *Atkinsoniella xanthonota* | MW533713 |
| Membracoidea | Cicadellidae | Cicadellinae | *Homalodisca vitripennis* | NC_006899 |
| Membracoidea | Cicadellidae | Cicadellinae | *Cofana yasumatsui* | NC_049087 |
| Membracoidea | Cicadellidae | Cicadellinae | *Bothrogonia qiongana* | NC_049894 |
| Membracoidea | Cicadellidae | Cicadellinae | *Bothrogonia tongmaiana* | NC_049895 |
| Membracoidea | Cicadellidae | Cicadellinae | *Bothrogonia yunana* | NC_049896 |
| Membracoidea | Cicadellidae | Coelidiinae | *Taharana fasciana* | NC_036015 |
| Membracoidea | Cicadellidae | Coelidiinae | *Olidiana ritcheriina* | NC_045207 |
| Membracoidea | Cicadellidae | Deltocephalinae | *Alobaldia tobae* | KY039116 |
| Membracoidea | Cicadellidae | Deltocephalinae | *Exitianus indicus* | KY039128 |
| Membracoidea | Cicadellidae | Deltocephalinae | *Orosius orientalis* | KY039146 |
| Membracoidea | Cicadellidae | Deltocephalinae | *Scaphoideus maai* | KY817243 |
| Membracoidea | Cicadellidae | Deltocephalinae | *Pellucidus guizhouensis* | MF784429 |
| Membracoidea | Cicadellidae | Deltocephalinae | *Drabescoides nuchalis* | NC_028154 |
| Membracoidea | Cicadellidae | Deltocephalinae | *Yanocephalus yanonis* | NC_036131 |
| Membracoidea | Cicadellidae | Deltocephalinae | *Maiestas dorsalis* | NC_036296 |
| Membracoidea | Cicadellidae | Deltocephalinae | *Japananus hyalinus* | NC_036298 |
| Membracoidea | Cicadellidae | Deltocephalinae | *Macrosteles quadrimaculatus* | NC_039560 |
| Membracoidea | Cicadellidae | Deltocephalinae | *Abrus expansivus* | NC_045238 |
| Membracoidea | Cicadellidae | Evacanthinae | *Evacanthus heimianus* | MG813486 |
| Membracoidea | Cicadellidae | Evacanthinae | *Evacanthus acuminatus* | MK948205 |
| Membracoidea | Cicadellidae | Hylicinae | *Kalasha nativa* | MW218662 |
| Membracoidea | Cicadellidae | Hylicinae | *Nacolus tuberculatus* | MW218663 |
| Membracoidea | Cicadellidae | Iassinae | *Batracomorphus lateprocessus* | MG813489 |
| Membracoidea | Cicadellidae | Iassinae | *Iassus dorsalis* | MN577634 |
| Membracoidea | Cicadellidae | Iassinae | *Trocnadella arisana* | NC_036480 |
| Membracoidea | Cicadellidae | Idiocerinae | *Idioscopus clypealis* | NC_039642 |
| Membracoidea | Cicadellidae | Idiocerinae | *Idiocerus salicis* | NC_046048 |
| Membracoidea | Cicadellidae | Ledrinae | *Ledra auditura* | MK387845 |
| Membracoidea | Cicadellidae | Ledrinae | *Tituria pyramidata* | NC_046701 |
| Membracoidea | Cicadellidae | Macropsinae | *Oncopsis nigrofasciata* | MG813492 |
| Membracoidea | Cicadellidae | Macropsinae | *Macropsis notata* | NC_042723 |
| Membracoidea | Cicadellidae | Megophthalminae | *Durgades nigropicta* | NC_035684 |
| Membracoidea | Cicadellidae | Megophthalminae | *Japanagallia spinosa* | NC_035685 |
| Membracoidea | Cicadellidae | Mileewinae | *Mileewa mira* | This study |
| Membracoidea | Cicadellidae | Mileewinae | *Mileewa lamellata* | This study |
| Membracoidea | Cicadellidae | Mileewinae | *Mileewa sharpa* | This study |
| Membracoidea | Cicadellidae | Mileewinae | *Mileewa amplimacula* | This study |
| Membracoidea | Cicadellidae | Mileewinae | *Processina sexmaculata* | This study |
| Membracoidea | Cicadellidae | Mileewinae | *Mileewa alara* | MW533151 |
| Membracoidea | Cicadellidae | Mileewinae | *Mileewa albovittata* | MK138358 |
| Membracoidea | Cicadellidae | Mileewinae | *Mileewa margheritae* | MT483998 |
| Membracoidea | Cicadellidae | Mileewinae | *Mileewa ponta* | MT497465 |
| Membracoidea | Cicadellidae | Mileewinae | *Ujna puerana* | MZ326688 |
| Membracoidea | Cicadellidae | Mileewinae | *Mileewa rufivena* | MZ326689 |
| Membracoidea | Membracidae | Smiliinae | *Entylia carinata* | NC_033539 |
| Membracoidea | Cicadellidae | Typhlocybinae | *Bolanusoides shaanxiensis* | MN661136 |
| Membracoidea | Cicadellidae | Typhlocybinae | *Parathailocyba orla* | MN894531 |
| Membracoidea | Cicadellidae | Typhlocybinae | *Eupteryx minuscula* | MN910279 |
| Membracoidea | Cicadellidae | Typhlocybinae | *Mitjaevia shibingensis* | MT981879 |
| Membracoidea | Cicadellidae | Typhlocybinae | *Limassolla lingchuanensis* | NC_046037 |
| Membracoidea | Cicadellidae | Typhlocybinae | *Empoascanara sipra* | NC_048516 |
| Membracoidea | Cicadellidae | Typhlocybinae | *Zyginella minuta* | NC_052876 |

Table S3. The best partition schemes and substitution models used in Bayesian inference (BI) and Maximum likelihood (ML) phylogenetic analyses.

|  | Subset | Best Model | Partition names |
| --- | --- | --- | --- |
| PCGs-BI | 1 | GTR+I+G | atp6_codon1 |
|  | 2 | GTR+I+G | atp6_codon2 |
|  | 3 | HKY+I+G | atp6_codon3 |
|  | 4 | GTR+I+G | atp8_codon1 |
|  | 5 | GTR+I+G | atp8_codon2, nad6_codon2 |
|  | 6 | GTR+I+G | atp8_codon3 |
|  | 7 | GTR+I+G | cox1_codon1 |
|  | 8 | GTR+I+G | cox1_codon2 |
|  | 9 | GTR+G | cox1_codon3 |
|  | 10 | GTR+I+G | cox2_codon1, cox3_codon1 |
|  | 11 | GTR+I+G | cytb_codon2, cox2_codon2 |
|  | 12 | GTR+G | cox2_codon3, cox3_codon3 |
|  | 13 | GTR+I+G | cox3_codon2 |
|  | 14 | GTR+I+G | cytb_codon1 |
|  | 15 | GTR+G | cytb_codon3 |
|  | 16 | GTR+I+G | nad1_codon1 |
|  | 17 | GTR+I+G | nad1_codon2, nad4L_codon2 |
|  | 18 | GTR+G | nad1_codon3 |
|  | 19 | GTR+I+G | nad2_codon1, nad3_codon1 |
|  | 20 | GTR+I+G | nad2_codon2 |
|  | 21 | GTR+G | nad2_codon3 |
|  | 22 | GTR+G | nad3_codon2 |
|  | 23 | GTR+G | nad3_codon3, nad6_codon3 |
|  | 24 | GTR+I+G | nad4L_codon1 |
|  | 25 | GTR+G | nad4_codon3, nad5_codon3, nad4L_codon3 |
|  | 26 | GTR+I+G | nad4_codon1 |
|  | 27 | GTR+I+G | nad4_codon2 |
|  | 28 | GTR+I+G | nad5_codon1 |
|  | 29 | GTR+I+G | nad5_codon2 |
|  | 30 | GTR+I+G | nad6_codon1 |
| PCGs-ML | 1 | GTR+F+R5 | atp6_codon1 |
|  | 2 | TVM+F+I+G4 | atp6_codon2, atp8_codon2, nad2_codon2, nad3_codon2, nad6_codon2 |
|  | 3 | TIM3+F+R5 | atp6_codon3, atp8_codon3, cytb_codon3, nad6_codon3 |
|  | 4 | TIM+F+R4 | atp8_codon1, nad2_codon1, nad3_codon1, nad6_codon1 |
|  | 5 | GTR+F+R5 | cox1_codon1, cytb_codon1 |
|  | 6 | GTR+F+R4 | cox1_codon2, cox2_codon2, cox3_codon2, cytb_codon2 |
|  | 7 | HKY+F+ASC+R5 | cox1_codon3, cox2_codon3,, cox3_codon3, nad3_codon3 |
|  | 8 | GTR+F+I+G4 | cox2_codon1, cox3_codon1 |
|  | 9 | GTR+F+R5 | nad1_codon1, nad4L_codon1, nad4_codon1, nad5_codon1 |
|  | 10 | GTR+F+I+G4 | nad1_codon2, nad4L_codon2, nad4_codon2, nad5_codon2 |
|  | 11 | TPM2+F+R6 | nad1_codon3, nad4L_codon3, nad4_codon3, nad5_codon3 |
|  | 12 | TN+F+ASC+R4 | nad2_codon3 |

Table S4. Codon number and Relative synonymous codon usage (RSCU) values in protein-coding genes (PCGs) of the five Mileewinae species mitogenomes.

| **A: *M. mira*** | |  |  |  |  |  |  |  |  |  |  |
| --- | --- | --- | --- | --- | --- | --- | --- | --- | --- | --- | --- |
| Codon | Count | RSCU | Codon | Count | RSCU | Codon | Count | RSCU | Codon | Count | RSCU |
| UUU(F) | 355 | 1.79 | UCU(S) | 124 | 2.6 | UAU(Y) | 145 | 1.64 | UGU(C) | 53 | 1.77 |
| UUC(F) | 41 | 0.21 | UCC(S) | 16 | 0.34 | UAC(Y) | 32 | 0.36 | UGC(C) | 7 | 0.23 |
| UUA(L) | 392 | 4.58 | UCA(S) | 107 | 2.25 | UAA(*) | 9 | 1.8 | UGA(W) | 77 | 1.67 |
| UUG(L) | 47 | 0.55 | UCG(S) | 7 | 0.15 | UAG(*) | 1 | 0.2 | UGG(W) | 15 | 0.33 |
| CUU(L) | 46 | 0.54 | CCU(P) | 67 | 2.31 | CAU(H) | 52 | 1.62 | CGU(R) | 19 | 1.52 |
| CUC(L) | 1 | 0.01 | CCC(P) | 7 | 0.24 | CAC(H) | 12 | 0.38 | CGC(R) | 1 | 0.08 |
| CUA(L) | 26 | 0.3 | CCA(P) | 38 | 1.31 | CAA(Q) | 45 | 1.76 | CGA(R) | 27 | 2.16 |
| CUG(L) | 1 | 0.01 | CCG(P) | 4 | 0.14 | CAG(Q) | 6 | 0.24 | CGG(R) | 3 | 0.24 |
| AUU(I) | 412 | 1.88 | ACU(T) | 62 | 1.86 | AAU(N) | 161 | 1.69 | AGU(S) | 30 | 0.63 |
| AUC(I) | 26 | 0.12 | ACC(T) | 5 | 0.15 | AAC(N) | 30 | 0.31 | AGC(S) | 3 | 0.06 |
| AUA(M) | 267 | 1.76 | ACA(T) | 65 | 1.95 | AAA(K) | 116 | 1.81 | AGA(S) | 92 | 1.93 |
| AUG(M) | 36 | 0.24 | ACG(T) | 1 | 0.03 | AAG(K) | 12 | 0.19 | AGG(S) | 2 | 0.04 |
| GUU(V) | 83 | 2.21 | GCU(A) | 45 | 2.17 | GAU(D) | 53 | 1.8 | GGU(G) | 79 | 1.79 |
| GUC(V) | 9 | 0.24 | GCC(A) | 4 | 0.19 | GAC(D) | 6 | 0.2 | GGC(G) | 1 | 0.02 |
| GUA(V) | 46 | 1.23 | GCA(A) | 31 | 1.49 | GAA(E) | 67 | 1.7 | GGA(G) | 61 | 1.38 |
| GUG(V) | 12 | 0.32 | GCG(A) | 3 | 0.14 | GAG(E) | 12 | 0.3 | GGG(G) | 36 | 0.81 |
| Average# codons=3651 | | |  |  |  |  |  |  |  |  |  |
|  |  |  |  |  |  |  |  |  |  |  |  |
| **B: *M. lamellata*** | |  |  |  |  |  |  |  |  |  |  |
| Codon | Count | RSCU | Codon | Count | RSCU | Codon | Count | RSCU | Codon | Count | RSCU |
| UUU(F) | 375 | 1.89 | UCU(S) | 111 | 2.4 | UAU(Y) | 157 | 1.78 | UGU(C) | 55 | 1.9 |
| UUC(F) | 21 | 0.11 | UCC(S) | 9 | 0.19 | UAC(Y) | 19 | 0.22 | UGC(C) | 3 | 0.1 |
| UUA(L) | 395 | 4.86 | UCA(S) | 129 | 2.79 | UAA(*) | 10 | 2 | UGA(W) | 79 | 1.76 |
| UUG(L) | 29 | 0.36 | UCG(S) | 2 | 0.04 | UAG(*) | 0 | 0 | UGG(W) | 11 | 0.24 |
| CUU(L) | 38 | 0.47 | CCU(P) | 63 | 2.07 | CAU(H) | 56 | 1.78 | CGU(R) | 23 | 1.84 |
| CUC(L) | 3 | 0.04 | CCC(P) | 5 | 0.16 | CAC(H) | 7 | 0.22 | CGC(R) | 0 | 0 |
| CUA(L) | 20 | 0.25 | CCA(P) | 49 | 1.61 | CAA(Q) | 48 | 1.88 | CGA(R) | 25 | 2 |
| CUG(L) | 3 | 0.04 | CCG(P) | 5 | 0.16 | CAG(Q) | 3 | 0.12 | CGG(R) | 2 | 0.16 |
| AUU(I) | 430 | 1.92 | ACU(T) | 65 | 1.88 | AAU(N) | 172 | 1.67 | AGU(S) | 24 | 0.52 |
| AUC(I) | 19 | 0.08 | ACC(T) | 3 | 0.09 | AAC(N) | 34 | 0.33 | AGC(S) | 1 | 0.02 |
| AUA(M) | 300 | 1.86 | ACA(T) | 69 | 2 | AAA(K) | 120 | 1.89 | AGA(S) | 92 | 1.99 |
| AUG(M) | 23 | 0.14 | ACG(T) | 1 | 0.03 | AAG(K) | 7 | 0.11 | AGG(S) | 2 | 0.04 |
| GUU(V) | 89 | 2.54 | GCU(A) | 39 | 2 | GAU(D) | 48 | 1.66 | GGU(G) | 67 | 1.49 |
| GUC(V) | 5 | 0.14 | GCC(A) | 3 | 0.15 | GAC(D) | 10 | 0.34 | GGC(G) | 2 | 0.04 |
| GUA(V) | 42 | 1.2 | GCA(A) | 35 | 1.79 | GAA(E) | 68 | 1.79 | GGA(G) | 93 | 2.07 |
| GUG(V) | 4 | 0.11 | GCG(A) | 1 | 0.05 | GAG(E) | 8 | 0.21 | GGG(G) | 18 | 0.4 |
| Average# codons=3649 | | |  |  |  |  |  |  |  |  |  |
|  |  |  |  |  |  |  |  |  |  |  |  |
| **C: *M. sharpa*** | |  |  |  |  |  |  |  |  |  |  |
| Codon | Count | RSCU | Codon | Count | RSCU | Codon | Count | RSCU | Codon | Count | RSCU |
| UUU(F) | 335 | 1.83 | UCU(S) | 108 | 2.39 | UAU(Y) | 160 | 1.69 | UGU(C) | 51 | 1.73 |
| UUC(F) | 31 | 0.17 | UCC(S) | 11 | 0.24 | UAC(Y) | 29 | 0.31 | UGC(C) | 8 | 0.27 |
| UUA(L) | 382 | 4.43 | UCA(S) | 126 | 2.78 | UAA(*) | 8 | 1.45 | UGA(W) | 72 | 1.64 |
| UUG(L) | 44 | 0.51 | UCG(S) | 7 | 0.15 | UAG(*) | 3 | 0.55 | UGG(W) | 16 | 0.36 |
| CUU(L) | 49 | 0.57 | CCU(P) | 55 | 1.93 | CAU(H) | 55 | 1.75 | CGU(R) | 13 | 1.06 |
| CUC(L) | 3 | 0.03 | CCC(P) | 7 | 0.25 | CAC(H) | 8 | 0.25 | CGC(R) | 4 | 0.33 |
| CUA(L) | 34 | 0.39 | CCA(P) | 46 | 1.61 | CAA(Q) | 45 | 1.8 | CGA(R) | 29 | 2.37 |
| CUG(L) | 5 | 0.06 | CCG(P) | 6 | 0.21 | CAG(Q) | 5 | 0.2 | CGG(R) | 3 | 0.24 |
| AUU(I) | 375 | 1.83 | ACU(T) | 69 | 1.73 | AAU(N) | 171 | 1.74 | AGU(S) | 23 | 0.51 |
| AUC(I) | 35 | 0.17 | ACC(T) | 11 | 0.28 | AAC(N) | 26 | 0.26 | AGC(S) | 5 | 0.11 |
| AUA(M) | 257 | 1.71 | ACA(T) | 76 | 1.9 | AAA(K) | 102 | 1.76 | AGA(S) | 63 | 1.39 |
| AUG(M) | 43 | 0.29 | ACG(T) | 4 | 0.1 | AAG(K) | 14 | 0.24 | AGG(S) | 19 | 0.42 |
| GUU(V) | 102 | 2.18 | GCU(A) | 53 | 2.44 | GAU(D) | 57 | 1.84 | GGU(G) | 85 | 1.86 |
| GUC(V) | 7 | 0.15 | GCC(A) | 4 | 0.18 | GAC(D) | 5 | 0.16 | GGC(G) | 7 | 0.15 |
| GUA(V) | 73 | 1.56 | GCA(A) | 28 | 1.29 | GAA(E) | 68 | 1.66 | GGA(G) | 73 | 1.6 |
| GUG(V) | 5 | 0.11 | GCG(A) | 2 | 0.09 | GAG(E) | 14 | 0.34 | GGG(G) | 18 | 0.39 |
| Average# codons=3652 | | |  |  |  |  |  |  |  |  |  |
|  |  |  |  |  |  |  |  |  |  |  |  |
| **D: *M. amplimacula*** | |  |  |  |  |  |  |  |  |  |  |
| Codon | Count | RSCU | Codon | Count | RSCU | Codon | Count | RSCU | Codon | Count | RSCU |
| UUU(F) | 333 | 1.82 | UCU(S) | 90 | 2.07 | UAU(Y) | 145 | 1.56 | UGU(C) | 59 | 1.82 |
| UUC(F) | 33 | 0.18 | UCC(S) | 15 | 0.34 | UAC(Y) | 41 | 0.44 | UGC(C) | 6 | 0.18 |
| UUA(L) | 350 | 4.27 | UCA(S) | 114 | 2.62 | UAA(*) | 10 | 1.82 | UGA(W) | 77 | 1.69 |
| UUG(L) | 55 | 0.67 | UCG(S) | 11 | 0.25 | UAG(*) | 1 | 0.18 | UGG(W) | 14 | 0.31 |
| CUU(L) | 46 | 0.56 | CCU(P) | 57 | 1.93 | CAU(H) | 49 | 1.51 | CGU(R) | 20 | 1.6 |
| CUC(L) | 1 | 0.01 | CCC(P) | 8 | 0.27 | CAC(H) | 16 | 0.49 | CGC(R) | 1 | 0.08 |
| CUA(L) | 36 | 0.44 | CCA(P) | 49 | 1.66 | CAA(Q) | 43 | 1.65 | CGA(R) | 26 | 2.08 |
| CUG(L) | 4 | 0.05 | CCG(P) | 4 | 0.14 | CAG(Q) | 9 | 0.35 | CGG(R) | 3 | 0.24 |
| AUU(I) | 387 | 1.8 | ACU(T) | 68 | 1.72 | AAU(N) | 162 | 1.63 | AGU(S) | 50 | 1.15 |
| AUC(I) | 43 | 0.2 | ACC(T) | 10 | 0.25 | AAC(N) | 37 | 0.37 | AGC(S) | 3 | 0.07 |
| AUA(M) | 293 | 1.75 | ACA(T) | 78 | 1.97 | AAA(K) | 110 | 1.75 | AGA(S) | 57 | 1.31 |
| AUG(M) | 41 | 0.25 | ACG(T) | 2 | 0.05 | AAG(K) | 16 | 0.25 | AGG(S) | 8 | 0.18 |
| GUU(V) | 77 | 1.95 | GCU(A) | 53 | 2.68 | GAU(D) | 55 | 1.67 | GGU(G) | 70 | 1.56 |
| GUC(V) | 8 | 0.2 | GCC(A) | 2 | 0.1 | GAC(D) | 11 | 0.33 | GGC(G) | 8 | 0.18 |
| GUA(V) | 64 | 1.62 | GCA(A) | 23 | 1.16 | GAA(E) | 69 | 1.84 | GGA(G) | 63 | 1.4 |
| GUG(V) | 9 | 0.23 | GCG(A) | 1 | 0.05 | GAG(E) | 6 | 0.16 | GGG(G) | 39 | 0.87 |
| Average# codons=3649 | | |  |  |  |  |  |  |  |  |  |
|  |  |  |  |  |  |  |  |  |  |  |  |
| **E: *P. sexmaculata*** | |  |  |  |  |  |  |  |  |  |  |
| Codon | Count | RSCU | Codon | Count | RSCU | Codon | Count | RSCU | Codon | Count | RSCU |
| UUU(F) | 332 | 1.84 | UCU(S) | 128 | 2.67 | UAU(Y) | 157 | 1.69 | UGU(C) | 53 | 1.93 |
| UUC(F) | 28 | 0.16 | UCC(S) | 14 | 0.29 | UAC(Y) | 29 | 0.31 | UGC(C) | 2 | 0.07 |
| UUA(L) | 355 | 4.23 | UCA(S) | 107 | 2.23 | UAA(*) | 10 | 1.82 | UGA(W) | 82 | 1.74 |
| UUG(L) | 48 | 0.57 | UCG(S) | 7 | 0.15 | UAG(*) | 1 | 0.18 | UGG(W) | 12 | 0.26 |
| CUU(L) | 43 | 0.51 | CCU(P) | 50 | 1.68 | CAU(H) | 48 | 1.45 | CGU(R) | 16 | 1.25 |
| CUC(L) | 3 | 0.04 | CCC(P) | 12 | 0.4 | CAC(H) | 18 | 0.55 | CGC(R) | 1 | 0.08 |
| CUA(L) | 51 | 0.61 | CCA(P) | 55 | 1.85 | CAA(Q) | 44 | 1.66 | CGA(R) | 28 | 2.2 |
| CUG(L) | 4 | 0.05 | CCG(P) | 2 | 0.07 | CAG(Q) | 9 | 0.34 | CGG(R) | 6 | 0.47 |
| AUU(I) | 364 | 1.8 | ACU(T) | 64 | 1.61 | AAU(N) | 151 | 1.61 | AGU(S) | 36 | 0.75 |
| AUC(I) | 41 | 0.2 | ACC(T) | 6 | 0.15 | AAC(N) | 36 | 0.39 | AGC(S) | 4 | 0.08 |
| AUA(M) | 305 | 1.85 | ACA(T) | 86 | 2.16 | AAA(K) | 99 | 1.74 | AGA(S) | 82 | 1.71 |
| AUG(M) | 24 | 0.15 | ACG(T) | 3 | 0.08 | AAG(K) | 15 | 0.26 | AGG(S) | 5 | 0.1 |
| GUU(V) | 91 | 2.09 | GCU(A) | 34 | 1.72 | GAU(D) | 53 | 1.61 | GGU(G) | 85 | 1.84 |
| GUC(V) | 7 | 0.16 | GCC(A) | 10 | 0.51 | GAC(D) | 13 | 0.39 | GGC(G) | 6 | 0.13 |
| GUA(V) | 68 | 1.56 | GCA(A) | 34 | 1.72 | GAA(E) | 54 | 1.59 | GGA(G) | 68 | 1.47 |
| GUG(V) | 8 | 0.18 | GCG(A) | 1 | 0.05 | GAG(E) | 14 | 0.41 | GGG(G) | 26 | 0.56 |
| Average# codons=3648 | | |  |  |  |  |  |  |  |  |  |
